# Prospective echocardiographic characterization of postnatal cardiac development in C57BL/6 mice: Strain analysis between birth and adulthood

**DOI:** 10.14814/phy2.71113

**Published:** 2026-09-24

**Authors:** Sehjin Jo, George Spyropoulos, Logan G. Hart, Taylor A. Covington, Arvind K. Pandey, Thomas Michel, Fotios Spyropoulos

**Affiliations:** ^1^ Division of Newborn Medicine, Brigham and Women's Hospital Harvard Medical School Boston Massachusetts USA; ^2^ Cardiac Critical Care Medicine Division, Children's Hospital of Philadelphia University of Pennsylvania Philadelphia Pennsylvania USA; ^3^ Cardiovascular Medicine Division, Brigham and Women's Hospital, Harvard Medical School Harvard University Boston Boston Massachusetts USA

**Keywords:** C57BL/6, echocardiography, myocardial strain, postnatal development, sex‐dependent differences

## Abstract

Echocardiography is critical for characterizing murine models of cardiovascular disease, yet normative data for the early postnatal period, particularly advanced functional metrics like myocardial strain, remain undefined. This developmental window features rapid cardiac maturation and is central to modeling congenital and neonatal heart disease. To establish normative, sex‐specific trajectories of cardiac structure and function between birth and adulthood, we performed prospective high‐frequency echocardiography in C57BL/6 mice at postnatal day 0 (P0), P8, P21, and week 8 (Wk8). Somatic growth and left ventricular structural dimensions increased progressively from P0 to Wk8. This structural maturation was accompanied by lower ejection fraction and fractional shortening, attributable to geometric remodeling. In contrast, speckle‐tracking strain analysis revealed that intrinsic myocardial deformation, including global circumferential and radial strain, remained stable throughout postnatal development. Importantly, we identified sexual dimorphism emerging during late adolescence; by Wk8, males exhibited significantly greater longitudinal and radial strain magnitudes than females. This study provides the first prospective, myocardial strain‐based echocardiographic characterization of murine cardiac development between birth and adulthood, identifying P21–Wk8 as a critical window for sex‐specific cardiac mechanics. These normative trajectories provide a translational framework for interpreting cardiac phenotypes in preclinical models of congenital, pediatric, and developmental cardiovascular disease.

## 1 INTRODUCTION

Mice have become a cornerstone of modern biomedical research due to their genetic modifiability, rapid life cycle, and translatability to human health. They have become indispensable model organisms, particularly in cardiovascular research. Given that mice have similar cardiac development sequences to humans (Krishnan et al., 2014), mouse models of heart disease have become increasingly prominent, and technology has similarly adapted to allow the use of the most common human diagnostic modalities such as echocardiography. As a non‐invasive, real‐time imaging platform that allows for comprehensive assessment of cardiac morphology and physiology, echocardiography carries many advantages when investigating phenotypic changes in models of heart disease including access to a wide range of parameters, high temporal resolution, and low cost (Gao et al., 2011; Lindsey et al., 2018). High‐frequency commercially available ultrasound systems (e.g., Vevo F2) can capture murine cardiac images with exceptional spatial and temporal resolution even at heart rates of 400–600 bpm. Furthermore, recent advances in speckle‐tracking technology (Bauer et al., 2011) have enabled strain analysis—a sensitive measure of intrinsic myocardial deformation that detects functional changes often missed by conventional B‐mode or M‐mode imaging.

Despite the widespread use of echocardiography, prospective normative data for murine cardiac development remain limited, in contrast to the abundant literature on adult models (Koch et al., 2013; Lindsey et al., 2018; Popp et al., 2025). Similar normative studies have been performed in other small‐animal models, including hamsters, where conventional and speckle‐tracking echocardiography have been used to establish normative cardiac intervals (Barros Filho et al., 2021). While fetal cardiac function has been characterized in C57BL/6 mice using in utero echocardiography, including assessments of ejection fraction, E/A ratio, and chamber dimensions (Yu et al., 2008), these fetal data do not extend into the immediate postnatal period. The transition from late gestation to early neonatal life is marked by rapid non‐linear changes during murine postnatal development, including ventricular wall thickening, chamber remodeling, and maturation of systolic and diastolic function (Le & Wagenseil, 2012; Wiesmann et al., 2000). As a result, normative data derived from fetal or adult stages cannot be extrapolated to neonatal or juvenile stages, potentially leading to misinterpretation of phenotypic severity or therapeutic efficacy (Spyropoulos et al., 2024; Zhang et al., 2021). In genetically derived disease models, baseline values are routinely provided to account for potential off‐target effects of genome editing and to enable comparison with the values observed in the disease of interest. Similarly, having a set of normal baseline values for wild‐type (WT) mice allows for testing different therapeutic options across preclinical models, identifying and quantifying disease establishment and progression at multiple time‐points, and monitoring therapeutic interventions prospectively. This approach can significantly reduce animal usage and study costs. Therefore, the purpose of this study is to characterize transthoracic echocardiography measurements and calculated parameters using B‐mode, M‐mode, Doppler‐mode, and myocardial strain in different developmental timepoints including upon birth (P0), postnatal day 8 (P8), juvenile postnatal day 21 (P21), and adulthood at 8 weeks of age (Wk8) in C57BL/6 WT mice.

## 2 MATERIALS AND METHODS

### 2.1 Animal care and use

All animal procedures were performed in accordance with NIH guidelines for the care of laboratory mice and were approved by the Brigham and Women's Hospital (BWH) Center for Comparative Medicine and Institutional Animal Care and Use Committee (protocol 2016N000278). C57BL/6 mice (Jackson Laboratories, JL) were housed in a controlled environment maintained at 21°C ± 2°C with 35% humidity, with an artificial 12‐h light/12‐h dark cycle. Animals had unrestricted access to a standard rodent diet (5053) and drinking water.

### 2.2 Study design

Adult male and female C57BL/6 mice used for breeding were 8–10 weeks old at the time of pairing, and all dams were nulliparous. Timed pregnancies were established by pairing adult female and male mice in the evening. The presence of a vaginal plug the following morning was designated as embryonic day (E) 0.5 after which the male was removed. Pregnant dams were monitored until delivery at E19.5.

**FIGURE 1:**
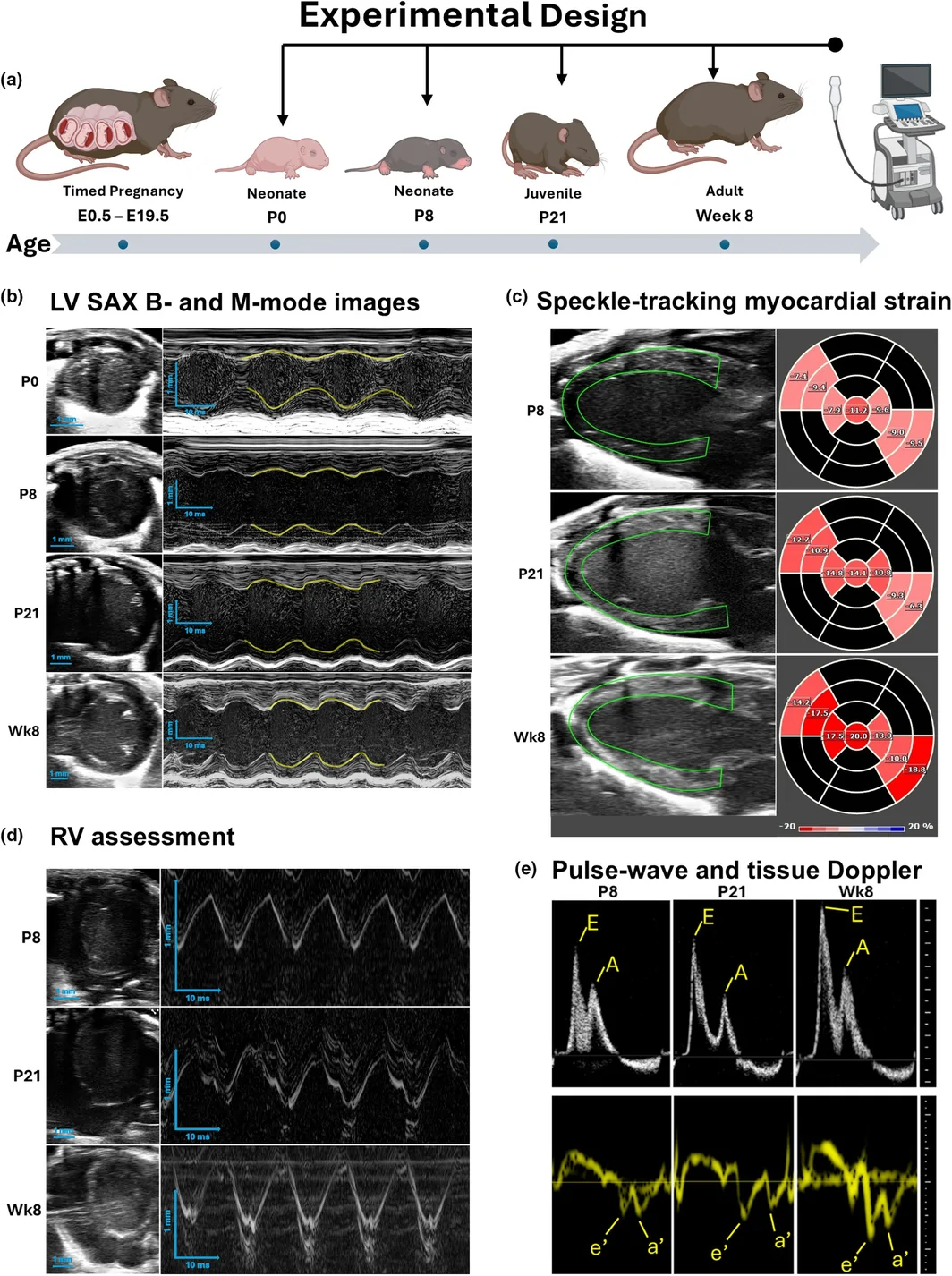
Experimental design and representative echocardiographic acquisition across postnatal development. (a) Schematic of the study design, illustrating timed pregnancy and serial echocardiographic assessments at postnatal Day 0 (P0), Day 8 (P8), Day 21 (P21), and Week 8 (Wk8). (b) Representative short axis (SAX) B‐mode (left) and corresponding M‐mode (right) images at P0, P8, P21, and Week 8. Blue bars indicate spatial (*y*‐axis, 1 mm) and temporal (*x*‐axis, 10 ms) scales. Yellow lines indicate chamber dimensions and wall motion. Note the progressive increase in chamber dimensions and wall thickness relative to the fixed spatial scale. Representative B‐mode echocardiography videos from P0 to Wk8 are included in the supplemental material (Supp. Movie S1–, S4). (c) Representative longitudinal strain contours (left; green outline) and the corresponding bullseye maps (right) derived from parasternal long‐axis views. The color scale (bottom right) ranges from −20% (red) to +20% (blue). Darker red segments in the bullseye maps indicate greater magnitude of peak systolic strain (inward displacement). The bullseye map segments correspond to the following anatomical regions from left to right: basal anteroseptal, mid anteroseptal, apical septal, apex, apical lateral, mid inferolateral and basal inferolateral. A video demonstration of the speckle‐tracking software interface and real‐time strain acquisition is provided in supplemental material (Supp. Movie S5–, S7). (d) Representative parasternal four‐chamber B‐mode images (left) and corresponding M‐mode tracings (right) illustrating tricuspid annular plane systolic excursion (TAPSE) assessment at P0, P8, P21, and week 8. (e) Representative pulse‐wave (PW) Doppler traces (top row) of transmitral inflow velocities, including the early diastolic filling wave (E) and late atrial contraction wave (A), and PW tissue doppler traces (bottom row) depicting myocardial velocities, including early diastolic relaxation (e') and late diastolic atrial contraction (a'), of transmitral inflow (top row) at P0, P8, P21, and Week 8. Vertical scales (right) indicate velocity (mm/s).

Pups underwent transthoracic echocardiography at birth (P0), at postnatal day 8 (P8), and at 3 weeks of age (P21). At P28, pups were weaned and separated by sex. Mice were then followed into adulthood for a final echocardiographic assessment at 8 weeks of age (Week 8, Wk8; see study design in Figure 1a). A total of 30 pups from three different litters were studied prospectively to adulthood (Figure 1a).

### 2.3 Transthoracic echocardiography

Echocardiography was performed at BWH using the VisualSonics Vevo F2 ultrasound system. The UHF71x (71–30 MHz) transducer was used in the neonatal cardiac preset from P0 to P21 and the UHF57x (57–25 MHz) transducer was used in the cardiac preset at Wk8.

For the P0 pups, no anesthesia or hair removal was used. Pups were gently restrained with surgical tape on the heated platform, and the tape was removed with adhesive remover after completing the study.

For P8‐Wk8 mice, anesthesia was induced with 2%–3% isoflurane mixed with 100% oxygen and then maintained at 1%–1.5% isoflurane to keep the heart rate above 400 bpm. Mice were positioned supine on a heated platform and gently secured with tape. Chest hair was removed using a depilatory cream and warmed ultrasound gel was applied. Isoflurane levels were adjusted throughout imaging to maintain target heart rates, and mice breathed spontaneously. Heart and respiratory rates were monitored continuously. Body temperature was maintained at 37°C using a heated platform, a gel warmer, and an overhead heat lamp.

### 2.4 Measurements

At P0, imaging included parasternal long axis (LAX) B‐mode, short axis (SAX) B‐mode, and SAX M‐mode views of the left ventricle (LV) (Spyropoulos et al., 2024). At least three consecutive cardiac cycles were obtained per cine loop with two to three cine loops recorded per imaging view for each animal. For each echocardiographic parameter, at least three measurements were performed and these measurements were averaged to obtain the final reported values. LV dimensions were obtained from parasternal short‐axis M‐mode images at the mid‐papillary level. All measurements were performed between breaths to avoid respiratory motion artifacts. Measurements included LV internal diameter (LVID), anterior wall thickness (AWT), and posterior wall thickness (PWT) in both systole (s) and diastole (d). LV end‐diastolic and end‐systolic volumes (LVEDV, LVESV) were calculated using the Teichholz formula: LVEDV = [7.0 × (LVIDd)^3^]/[2.4 + LVIDd] and LVESV = [7.0 × (LVIDs)^3^]/[2.4 + LVIDs]. Systolic function was derived from these volumes as follows: Stroke volume (SV) was calculated as the difference between end‐diastolic and end‐systolic volumes: SV (μL) = LVEDV − LVESV. Cardiac output (CO) was calculated as: CO (mL/min) = [SV (μL) × HR (bpm)]/1000. Ejection fraction (EF) = [(LVEDV − LVESV)/LVEDV] × 100 and Fractional shortening (FS) = [(LVIDd − LVIDs)/LVIDd] × 100.

At P0, the apical four‐chamber view could not be consistently obtained due to the extremely small cardiac size, limited acoustic windows, and the technical constraints of neonatal cardiac imaging. Consequently, mitral valve flow Doppler, tissue Doppler, speckle‐tracking strain analysis, and RV assessment were not performed at this time point. Conventional echocardiographic parameters were successfully obtained in all animals at all time points. From P8 onward, diastolic function was assessed using pulse‐wave Doppler and tissue Doppler imaging (TDI) obtained in the apical four‐chamber view. The transmitral inflow sample volume was positioned at the tip of the mitral leaflets to record maximal transmitral inflow velocities. Measured parameters included early transmitral flow velocity (E), late transmitral flow velocity (A), aortic ejection time (AET), mitral valve ejection time (ET), isovolumic contraction time (IVCT) and isovolumic relaxation time (IVRT). For TDI, a pulse‐wave Doppler sample volume was placed at the septal corner of the mitral annulus. Extracted measurements included peak early‐diastolic annular velocity (e’), peak late‐diastolic annular velocity (a’) and systolic annular velocity (s’). All Doppler recordings sampled at least three cardiac cycles per cine loop, and a minimum of three measurements per animal were analyzed and averaged. Waveforms were visually inspected with temporal alignment of transmitral and annular recordings to ensure correct peak identification.

Right ventricular (RV) function was assessed using M‐mode‐derived tricuspid annular plane systolic excursion (TAPSE), measured as the base‐to‐apex shortening of the RV during systole in the apical four‐chamber view (Spyropoulos et al., 2020). TAPSE was measured between breaths to minimize respiratory motion artifacts.

Left atrial (LA) dimensions were assessed from parasternal long‐axis B‐mode cine loops. Maximum LA area (LAA systole) was measured at end‐ventricular systole, corresponding to the end of the T wave on the ECG, immediately before mitral valve opening. Minimum LA area (LAA diastole) was measured at end‐ventricular diastole, corresponding to the QRS complex on the ECG, immediately after mitral valve closure. The endocardial border of the left atrium was manually traced at each frame to obtain LA area. LA fractional area change (LA FAC) was calculated as [(LAA systole − LAA diastole)/LAA systole] × 100. LA measurements were feasible from P8 onward; LA visualization could not be reliably obtained at P0 due to the technical constraints of neonatal imaging.

All views were digitally stored in cine loops consisting of 300 frames. The Vevo Lab Version 5.11.0 Cardiac Package was used to extract measurements from acquired images which were subsequently analyzed offline. Strain measurements were performed using parasternal LAX B‐mode loops and analyzed with Vevo Strain 2.0 (VisualSonics). Loops were accepted for analysis only when the endocardial and epicardial borders could be tracked continuously across two to three consecutive cardiac cycles; frames affected by respiratory motion were excluded before tracking. Endocardial and epicardial borders were manually traced and confirmed across two to three cardiac cycles to ensure optimal speckle tracking. End‐diastole was defined as the time of maximal LV cavity dimension and served as the reference point for the strain cycle. Myocardial strain values represent deformation across the full thickness of the LV wall (from endocardium to epicardium) (Movie S8), whereas endocardial strain values specifically quantify deformation along the endocardial contour (Movie S9), Using this approach, we quantified myocardial global circumferential strain (MyoGCS), myocardial global longitudinal strain (MyoGLS), endocardial global circumferential strain (EndoGCS), endocardial global longitudinal strain (EndoGLS), and global radial strain (GRS).

To assess methodological agreement between SAX M‐mode and LAX B‐mode derived volumetric parameters, a post‐hoc analysis was performed at all four developmental timepoints. Left ventricular end‐diastolic volume (LVEDV) and end‐systolic volume (LVESV) were measured from LAX B‐mode cine loops using Simpson's monoplane method. End‐diastolic and end‐systolic frames were identified by maximal and minimal LV cavity dimensions respectively, and the endocardial border was manually traced at each frame. Ejection fraction was calculated as [(LVEDV − LVESV)/LVEDV] × 100. These LAX B‐mode derived measurements were compared with the corresponding SAX M‐mode Teichholz‐derived values using Bland–Altman analysis as described in the Statistical Analysis section.

### 2.5 Statistical analysis

All statistical analyses were conducted using R statistical software (R version 4.5.2, Foundation for Statistical Computing, Vienna, Austria) with the following packages: rstatix, lmerTest, multcompView, and gt. Descriptive statistics are reported as mean ± standard deviation (SD). The normality of data distribution was evaluated using the Shapiro–Wilk test. To analyze developmental changes in echocardiographic and physiological parameters, a one‐way repeated measures analysis of variance (ANOVA) was performed. For parameters that did not meet normality assumptions, statistical conclusions were cross‐validated using the non‐parametric Friedman test followed by Bonferroni‐corrected Wilcoxon signed‐rank post‐hoc tests. Because the parametric and non‐parametric approaches yielded concordant results for all variables, parametric statistics are reported for consistency and clarity.

Left atrial parameters (systolic LA area, diastolic LA area, and LA fractional area change) were analyzed using linear mixed models with animal as a random effect (lmerTest package), as measurement feasibility was variable across timepoints, resulting in an unbalanced repeated measures design (Table S3). This approach retains all available observations rather than excluding animals with incomplete data.

Post‐hoc pairwise comparisons were conducted using Bonferroni‐corrected paired *t*‐tests. Significant differences between timepoints are indicated by superscript letters in summary tables.

To evaluate methodological agreement between SAX M‐mode and LAX B‐mode derived ejection fraction, a Bland–Altman analysis was performed. For each animal at each timepoint, the difference between LAX B‐mode and SAX M‐mode derived EF was plotted against the mean of the two methods. Bias was defined as the mean difference between methods, and the 95% limits of agreement were calculated as bias ±1.96 standard deviations.

To evaluate potential sexual dimorphism in cardiac maturation, a two‐way mixed ANOVA was employed with time as the within‐subjects factor and sex as the between‐subjects factor. Interaction terms (Time × Sex) were analyzed to identify sex‐specific developmental patterns. Direct comparisons between males and females at individual timepoints were performed using independent samples *t*‐tests. All hypothesis tests were two‐sided, and a *p*‐value of <0.05 was considered statistically significant. Parameters are described as stable across development when no significant effect of timepoint was detected by repeated‐measures ANOVA.

## 3 RESULTS

### 3.1 Serial echocardiographic assessment across postnatal development

A total of 30 wild‐type C57BL/6 mice from three litters were evaluated at P0, P8, P21, and Wk8. One litter was culled from 12 pups to 10 pups after P0 to standardize litter sizes (Kirsanov & Geyer, 2023). All animals successfully underwent echocardiography at all time points. Representative parasternal SAX and M‐mode images illustrating chamber enlargement and progressive myocardial thickening are shown in Figure 1b, representative strain images in Figure 1c, representative parasternal four‐chamber B‐mode images and corresponding M‐mode tracings in Figure 1d, while Doppler and tissue Doppler waveforms are shown in Figure 1e. Serial measurements obtained across all modalities are summarized in Table 1, with corresponding male‐only and female‐only datasets in Tables 2 and 3, respectively.

**TABLE 1:** Prospective analysis of echocardiographic parameters from birth to adulthood.

Parameter	P0 (*n* = 30)	P8 (*n* = 28)	P21 (*n* = 26)	Week 8 (*n* = 26)	*p*‐value
Sex	m (13)/f (17)	m (13)/f (15)	m (13)/f (13)	m (13)/f (13)	
BW (g)	1.25 ± 0.08^a^	3.78 ± 0.38^b^	7.27 ± 0.86^c^	20.79 ± 2.27^d^	<0.001***
*Short Axis M‐mode*					
HR (bpm)	325 ± 35^a^	475 ± 39^b^	416 ± 33^c^	441 ± 39^c^	<0.001***
dLVD (mm)	1.52 ± 0.15^a^	2.15 ± 0.18^b^	2.9 ± 0.21^c^	3.72 ± 0.19^d^	<0.001***
sLVD (mm)	0.98 ± 0.14^a^	1.43 ± 0.18^b^	1.94 ± 0.24^c^	2.54 ± 0.32^d^	<0.001***
dLVAW (mm)	0.23 ± 0.04^a^	0.52 ± 0.07^b^	0.54 ± 0.08^b^	0.7 ± 0.12^c^	<0.001***
sLVAW (mm)	0.44 ± 0.05^a^	0.79 ± 0.1^b^	0.88 ± 0.12^c^	1.09 ± 0.19^d^	<0.001***
dLVPW (mm)	0.25 ± 0.06^a^	0.45 ± 0.07^b^	0.49 ± 0.14^b^	0.58 ± 0.09^c^	<0.001***
sLVPW (mm)	0.47 ± 0.08^a^	0.69 ± 0.08^b^	0.77 ± 0.2^b^	0.95 ± 0.13^c^	<0.001***
LVEDV (μL)	6.39 ± 1.48^a^	15.39 ± 3.09^b^	32.44 ± 5.57^c^	59.22 ± 7.22^d^	<0.001***
LVESV (μL)	2.03 ± 0.66^a^	5.52 ± 1.7^b^	12.12 ± 3.6^c^	23.92 ± 7.36^d^	<0.001***
EF (%)	69 ± 5.44^a^	64.71 ± 5.66^b^	63.24 ± 6.81^bc^	60.29 ± 8.29^c^	<0.001***
FS (%)	35.8 ± 4.15^a^	33.41 ± 3.97^ab^	33.21 ± 4.98^ab^	31.84 ± 5.69^b^	0.017*
SV (μL)	4.36 ± 0.93^a^	9.87 ± 1.78^b^	20.32 ± 3.13^c^	35.31 ± 3.76^d^	<0.001***
CO (mL/min)	1.43 ± 0.38^a^	4.66 ± 0.74^b^	8.43 ± 1.26^c^	15.58 ± 2.31^d^	<0.001***
*Long Axis B‐mode*					
dLAA (mm^2^)		2 ± 0.26^a^	3.31 ± 0.58^b^	5.14 ± 0.93^c^	<0.001***
sLAA (mm^2^)		2.43 ± 0.29^a^	3.9 ± 0.6^b^	6.6 ± 1.06^c^	<0.001***
FAC (%)		17.48 ± 6.33^a^	14.9 ± 9.36^a^	22.11 ± 7.33^a^	0.011*
*Mitral valve doppler*					
a’ (mm/s)		13.97 ± 3.15^a^	18.08 ± 3.93^b^	24.15 ± 4.5^c^	<0.001***
e’ (mm/s)		12.09 ± 2.43^a^	22.92 ± 5.81^b^	25.25 ± 5.83^b^	<0.001***
s' (mm/s)		12.32 ± 1.31^a^	15.62 ± 2.42^b^	19.42 ± 3.5^c^	<0.001***
AET (ms)		49.64 ± 4.58^a^	52.35 ± 6.19^a^	51.53 ± 4.37^a^	0.044*
IVCT (ms)		15.39 ± 1.92^a^	15.95 ± 4.16^ab^	18 ± 3.12^b^	0.023*
IVRT (ms)		20.36 ± 1.77	19.11 ± 1.91	18.88 ± 3.12	0.06
ET (ms)		46.51 ± 8.16^a^	61 ± 8.5^b^	50.69 ± 7.65^a^	<0.001***
A (mm/s)		355.9 ± 50.3^a^	329.6 ± 56.4^a^	486.3 ± 95.8^b^	<0.001***
E (mm/s)		455.7 ± 69.2^a^	592.2 ± 58.3^b^	748.8 ± 79.4^c^	<0.001***
E/A		1.28 ± 0.16^a^	1.82 ± 0.22^b^	1.55 ± 0.28^c^	<0.001***
E/e’		38.92 ± 8.76^a^	27.03 ± 5.4^b^	30.67 ± 5.16^b^	<0.001***
*RV function*					
TAPSE (mm)		0.4 ± 0.06^a^	0.54 ± 0.07^b^	0.78 ± 0.12^c^	<0.001***
*Strain*					
MyoGCS (%)		−16.22 ± 3.75	−18.33 ± 3.25	−16.64 ± 4.34	0.12
MyoGLS (%)		−8.79 ± 2.64^a^	−11.88 ± 2.38^b^	−11.1 ± 2.54^b^	<0.001***
EndoGCS (%)		−28.77 ± 6.46	−30.66 ± 6.22	−28.72 ± 8.21	0.514
EndoGLS (%)		−14.13 ± 4.52	−16.9 ± 3.54	−15.36 ± 4.3	0.067
GRS (%)		49.36 ± 18.47	61.84 ± 18.19	53.41 ± 23.9	0.051

*Note*: Data are presented as mean ± standard deviation (SD). *p*‐values derived from one‐way repeated measures ANOVA. Superscript letters (a–d) indicate significant differences (*p* < 0.05) between time points based on Bonferroni‐corrected post‐hoc pairwise comparisons; time points sharing a letter are not significantly different. Asterisks denote overall ANOVA significance: *p < 0.05, **p < 0.01, ***p < 0.001. The prefixes ‘d’ and ‘s’ denote diastole and systole, respectively.

Abbreviations: A, late diastolic transmitral flow velocity; a’, peak late‐diastolic annular velocity; AET, aortic ejection time; BW, body weight; CO, cardiac output; E, early diastolic transmitral flow velocity; e’, peak early‐diastolic annular velocity; EF, left ventricular ejection fraction (Teichholz); EndoGCS, endocardial global circumferential strain; EndoGLS, endocardial global longitudinal strain; ET, mitral valve ejection time; f, female; FAC, left atrial fractional area change; FS, fractional shortening; GRS, global radial strain; HR, heart rate; IVCT, isovolumic contraction time; IVRT, isovolumic relaxation time; LAA, left atrial area; LVAW, left ventricular anterior wall thickness; LVD, left ventricular diameter; LVEDV, left ventricular end‐diastolic volume; LVESV, left ventricular end‐systolic volume; LVPW, left ventricular posterior wall thickness; m, male; MyoGCS, myocardial global circumferential strain; MyoGLS, myocardial global longitudinal strain; s', systolic annular velocity; SV, stroke volume; TAPSE, tricuspid annulus plane systolic excursion.

**TABLE 2:** Prospective echocardiographic trajectories in male C57BL/6 Mice.

Parameter	P0 (*n* = 13)	P8 (*n* = 13)	P21 (*n* = 13)	Week 8 (*n* = 13)	*p*‐value
BW (g)	1.22 ± 0.08^a^	3.72 ± 0.36^b^	7.17 ± 0.92^c^	22.75 ± 0.84^d^	<0.001***
*Short Axis M‐mode*					
HR (bpm)	316 ± 37^a^	467 ± 26^b^	408 ± 30^c^	439 ± 47^bc^	<0.001***
dLVD (mm)	1.49 ± 0.18^a^	2.12 ± 0.21^b^	2.86 ± 0.2^c^	3.74 ± 0.2^d^	<0.001***
sLVD (mm)	0.95 ± 0.16^a^	1.39 ± 0.2^b^	1.88 ± 0.23^c^	2.52 ± 0.33^d^	<0.001***
dLVAW (mm)	0.23 ± 0.03^a^	0.51 ± 0.06^b^	0.52 ± 0.08^b^	0.72 ± 0.11^c^	<0.001***
sLVAW (mm)	0.42 ± 0.05^a^	0.78 ± 0.1^b^	0.88 ± 0.13^b^	1.1 ± 0.19^c^	<0.001***
dLVPW (mm)	0.25 ± 0.07^a^	0.44 ± 0.07^b^	0.49 ± 0.1^b^	0.61 ± 0.11^c^	<0.001***
sLVPW (mm)	0.47 ± 0.08^a^	0.68 ± 0.07^b^	0.76 ± 0.15^b^	1.01 ± 0.13^c^	<0.001***
LVEDV (μL)	6.1 ± 1.64^a^	14.95 ± 3.64^b^	31.36 ± 5.09^c^	60.03 ± 7.67^d^	<0.001***
LVESV (μL)	1.86 ± 0.69^a^	5.15 ± 1.91^b^	11.15 ± 3.02^c^	23.31 ± 7.81^d^	<0.001***
EF (%)	70.46 ± 5.42^a^	66.26 ± 5.97^ab^	64.87 ± 6.83^ab^	61.88 ± 8.22^b^	0.001**
FS (%)	36.91 ± 4.33	34.52 ± 4.21	34.38 ± 5.23	32.96 ± 5.63	0.069
SV (μL)	4.24 ± 1.01^a^	9.8 ± 2.11^b^	20.21 ± 3.31^c^	36.72 ± 3.76^d^	<0.001***
CO (mL/min)	1.34 ± 0.37^a^	4.56 ± 0.93^b^	8.19 ± 1.07^c^	16.15 ± 2.68^d^	<0.001***
*Long Axis B‐mode*					
dLAA (mm^2^)		2.01 ± 0.32^a^	3.46 ± 0.59^b^	4.97 ± 1.01^b^	<0.001***
sLAA (mm^2^)		2.46 ± 0.36^a^	3.92 ± 0.66^b^	6.39 ± 1.25^c^	<0.001***
FAC (%)		18.51 ± 6.38^ab^	11.51 ± 8.8^a^	21.95 ± 7.74^b^	0.005**
*Mitral valve doppler*					
a’ (mm/s)		13.43 ± 2.88^a^	17.85 ± 3.98^b^	25.61 ± 5.29^c^	<0.001***
e’ (mm/s)		12.02 ± 2.56^a^	23.44 ± 5.91^b^	26.35 ± 6.61^b^	<0.001***
s' (mm/s)		12.33 ± 1.28^a^	15.86 ± 2.64^b^	21.52 ± 3.65^c^	<0.001***
AET (ms)		48.95 ± 4.77	51.21 ± 6.73	49.81 ± 3.69	0.510
IVCT (ms)		14.62 ± 1.48	16.8 ± 4.8	17.37 ± 2.95	0.102
IVRT (ms)		20.26 ± 1.74	19.22 ± 2.16	18.46 ± 3.78	0.250
ET (ms)		49.26 ± 7.98^a^	62.47 ± 7.24^b^	52.93 ± 8.35^ab^	<0.001***
A (mm/s)		361 ± 57.6^a^	332.3 ± 33^a^	501.1 ± 89.9^b^	<0.001***
E (mm/s)		460.5 ± 73.5^a^	597.4 ± 57.9^b^	759.6 ± 98.2^c^	<0.001***
E/A		1.28 ± 0.15^a^	1.81 ± 0.17^b^	1.5 ± 0.24^a^	<0.001***
E/e’		39.72 ± 9.76^a^	26.85 ± 6.31^b^	30.05 ± 5.87^b^	<0.001***
*RV function*					
TAPSE (mm)		0.39 ± 0.06^a^	0.55 ± 0.06^b^	0.81 ± 0.13^c^	<0.001***
*Strain*					
MyoGCS (%)		−16.64 ± 4.41	−17.94 ± 2.8	−17.68 ± 5.19	0.642
MyoGLS (%)		−8.42 ± 2.89^a^	−11.91 ± 2.29^b^	−12.24 ± 2.53^b^	0.001**
EndoGCS (%)		−29.03 ± 6.95	−29.56 ± 4.63	−31.17 ± 9.79	0.643
EndoGLS (%)		−13.88 ± 5.11^a^	−16.67 ± 3.3^a^	−17.82 ± 3.99^a^	0.034*
GRS (%)		46.95 ± 20.84^a^	58 ± 15.87^ab^	65.32 ± 21.91^b^	0.012*

*Note*: Data are presented as mean ± standard deviation (SD). *p*values derived from one‐way repeated measures ANOVA. Superscript letters (a–d) indicate significant differences (*p* < 0.05) between time points based on Bonferroni‐corrected post‐hoc pairwise comparisons; time points sharing a letter are not significantly different. Asterisks denote overall ANOVA significance: *p < 0.05, **p < 0.01, ***p < 0.001. The prefixes ‘d’ and ‘s’ denote diastole and systole, respectively.

Abbreviations: A, late diastolic transmitral flow velocity; a’, peak late‐diastolic annular velocity; AET, aortic ejection time; BW, body weight; CO, cardiac output; E, early diastolic transmitral flow velocity; e’, peak early‐diastolic annular velocity; EF, left ventricular ejection fraction (Teichholz); EndoGCS, endocardial global circumferential strain; EndoGLS, endocardial global longitudinal strain; ET, mitral valve ejection time; FAC, left atrial fractional area change; FS, fractional shortening; GRS, global radial strain; HR, heart rate; IVCT, isovolumic contraction time; IVRT, isovolumic relaxation time; LAA, left atrial area; LVAW, left ventricular anterior wall thickness; LVD, left ventricular diameter; LVEDV, left ventricular end‐diastolic volume; LVESV, left ventricular end‐systolic volume; LVPW, left ventricular posterior wall thickness; MyoGCS, myocardial global circumferential strain; MyoGLS, myocardial global longitudinal strain; s', systolic annular velocity; SV, stroke volume; TAPSE, tricuspid annulus plane systolic excursion.

**TABLE 3:** Prospective echocardiographic trajectories in female C57BL/6 mice.

Parameter	P0 (*n* = 17)	P8 (*n* = 15)	P21 (*n* = 13)	Week 8 (*n* = 13)	*p*‐value
BW (g)	1.26 ± 0.08^a^	3.83 ± 0.4^b^	7.38 ± 0.83^c^	18.82 ± 1.28^d^	<0.001***
*Short Axis M‐mode*					
HR (bpm)	331.9 ± 32.1^a^	482.4 ± 47.7^b^	424.6 ± 35.8^c^	442.6 ± 30.3^bc^	<0.001***
dLVD (mm)	1.54 ± 0.13^a^	2.17 ± 0.15^b^	2.94 ± 0.23^c^	3.7 ± 0.19^d^	<0.001***
sLVD (mm)	1.01 ± 0.12^a^	1.47 ± 0.15^b^	2 ± 0.25^c^	2.57 ± 0.32^d^	<0.001***
dLVAW (mm)	0.24 ± 0.04^a^	0.53 ± 0.08^b^	0.56 ± 0.08^b^	0.69 ± 0.14^c^	<0.001***
sLVAW (mm)	0.45 ± 0.05^a^	0.79 ± 0.1^b^	0.87 ± 0.1^b^	1.08 ± 0.19^c^	<0.001***
dLVPW (mm)	0.25 ± 0.06^a^	0.46 ± 0.07^b^	0.5 ± 0.17^b^	0.56 ± 0.07^b^	<0.001***
sLVPW (mm)	0.46 ± 0.08^a^	0.7 ± 0.1^b^	0.78 ± 0.24^bc^	0.89 ± 0.1^c^	<0.001***
LVEDV (μL)	6.6 ± 1.35^a^	15.77 ± 2.6^b^	33.53 ± 6.02^c^	58.42 ± 6.95^d^	<0.001***
LVESV (μL)	2.15 ± 0.62^a^	5.84 ± 1.48^b^	13.09 ± 3.98^c^	24.53 ± 7.14^d^	<0.001***
EF (%)	67.89 ± 5.35^a^	63.37 ± 5.21^a^	61.61 ± 6.65^a^	58.7 ± 8.38^a^	0.005**
FS (%)	34.96 ± 3.92	32.45 ± 3.61	32.04 ± 4.63	30.73 ± 5.76	0.099
SV (μL)	4.45 ± 0.88^a^	9.93 ± 1.5^b^	20.44 ± 3.08^c^	33.89 ± 3.33^d^	<0.001***
CO (mL/min)	1.49 ± 0.38^a^	4.75 ± 0.55^b^	8.67 ± 1.43^c^	15 ± 1.8^d^	<0.001***
*Long Axis B‐mode*					
dLAA (mm^2^)		2 ± 0.22^a^	3.16 ± 0.56^b^	5.33 ± 0.83^c^	<0.001***
sLAA (mm^2^)		2.4 ± 0.23^a^	3.88 ± 0.57^b^	6.83 ± 0.8^c^	<0.001***
FAC (%)		16.63 ± 6.44	18.29 ± 9.05	22.27 ± 7.22	0.370
*Mitral valve doppler*					
a’ (mm/s)		14.45 ± 3.39^a^	18.32 ± 4.03^ab^	22.42 ± 2.65^b^	<0.001***
e’ (mm/s)		12.16 ± 2.41^a^	22.36 ± 5.91^b^	24.16 ± 4.95^b^	<0.001***
s' (mm/s)		12.31 ± 1.37^a^	15.36 ± 2.24^b^	17.33 ± 1.62^b^	<0.001***
AET (ms)		50.24 ± 4.48^a^	53.59 ± 5.57^ab^	53.25 ± 4.44^b^	0.014*
IVCT (ms)		16.07 ± 2.04^a^	15.03 ± 3.3^a^	18.63 ± 3.27^a^	0.012*
IVRT (ms)		20.44 ± 1.85	18.98 ± 1.68	19.29 ± 2.36	0.127
ET (ms)		44.13 ± 7.8^a^	59.41 ± 9.76^b^	48.46 ± 6.44^a^	<0.001***
A (mm/s)		351.1 ± 44.1^a^	326.7 ± 75.8^a^	472.7 ± 103^b^	<0.001***
E (mm/s)		451.5 ± 67.7^a^	586.5 ± 60.8^b^	738.1 ± 57.1^c^	<0.001***
E/A		1.28 ± 0.17^a^	1.84 ± 0.27^b^	1.59 ± 0.31^b^	<0.001***
E/e’		38.22 ± 8.08^a^	27.22 ± 4.49^b^	31.29 ± 4.49^ab^	<0.001***
*RV function*					
TAPSE (mm)		0.41 ± 0.06^a^	0.53 ± 0.08^b^	0.76 ± 0.11^c^	<0.001***
*Strain*					
MyoGCS (%)		−15.86 ± 3.19	−18.72 ± 3.72	−15.61 ± 3.14	0.051
MyoGLS (%)		−9.12 ± 2.46^a^	−11.84 ± 2.56^a^	−9.96 ± 2.06^a^	0.026*
EndoGCS (%)		−28.54 ± 6.24	−31.77 ± 7.51	−26.26 ± 5.62	0.120
EndoGLS (%)		−14.35 ± 4.11^ab^	−17.13 ± 3.89^a^	−12.9 ± 3.09^b^	0.016*
GRS (%)		51.44 ± 16.61^ab^	65.69 ± 20.14^a^	41.51 ± 20.07^b^	0.011*

*Note*: Data are presented as mean ± standard deviation (SD). *p*‐values derived from one‐way repeated measures ANOVA. Superscript letters (a–d) indicate significant differences (*p* < 0.05) between time points based on Bonferroni‐corrected post‐hoc pairwise comparisons; time points sharing a letter are not significantly different. Asterisks denote overall ANOVA significance: *p < 0.05, **p < 0.01, ***p < 0.001. The prefixes ‘d’ and ‘s’ denote diastole and systole, respectively.

Abbreviations: A, late diastolic transmitral flow velocity; a’, peak late‐diastolic annular velocity; AET, aortic ejection time; BW, body weight; CO, cardiac output; E, early diastolic transmitral flow velocity; e’, peak early‐diastolic annular velocity; EF, left ventricular ejection fraction (Teichholz); EndoGCS, endocardial global circumferential strain; EndoGLS, endocardial global longitudinal strain; ET, mitral valve ejection time; FAC, left atrial fractional area change; FS, fractional shortening; GRS, global radial strain; HR, heart rate; IVCT, isovolumic contraction time; IVRT, isovolumic relaxation time; LAA, left atrial area; LVAW, left ventricular anterior wall thickness; LVD, left ventricular diameter; LVEDV, left ventricular end‐diastolic volume; LVESV, left ventricular end‐systolic volume; LVPW, left ventricular posterior wall thickness; MyoGCS, myocardial global circumferential strain; MyoGLS, myocardial global longitudinal strain; s', systolic annular velocity; SV, stroke volume; TAPSE, tricuspid annulus plane systolic excursion.

### 3.2 Somatic growth and structural cardiac maturation

**FIGURE 2:**
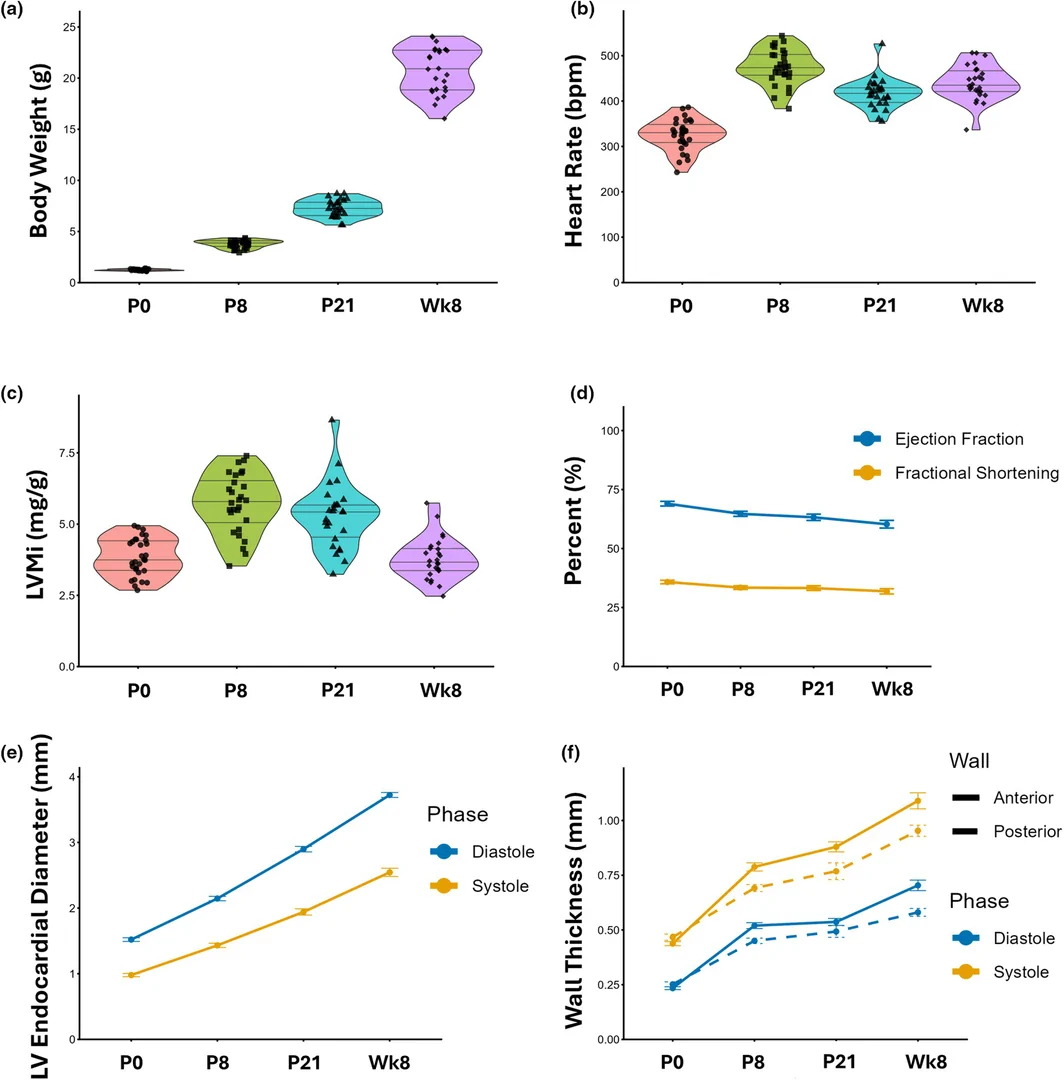
Postnatal Trajectories of Somatic Growth and Cardiac Function. (a–c) Violin plots displaying developmental changes in: (a) Body weight, (b) heart rate, and (c) left ventricle (LV) mass index. (d–f) Line graphs illustrate developmental trajectories for: (d) Global systolic function (ejection fraction [EF] and fractional shortening [FS]), (e) LV endocardial diameter, and (f) LV wall thickness. Data presented as violin plots with individual data points or mean ± SEM. Statistical significance for developmental comparisons is reported in the corresponding tables; figures are shown to illustrate developmental trajectories.

Body weight increased significantly between each developmental timepoint (*p* < 0.001), rising from 1.25 ± 0.08 g at P0 to 20.79 ± 2.27 g at Wk8 (Figure 2a, Table 1). LV dimensions, including diastolic/systolic diameters and wall thickness, increased in parallel. LVEDV and LVESV also rose progressively (*p* < 0.001), reflecting increasing chamber size with growth. Body‐weight–indexed LV mass (LVMi) increased from P0 to P8 and remained elevated at P21 before declining toward early‐postnatal values at Wk8 (Figure 2c). Progressive changes in LV geometry and wall motion are visually evident in the representative SAX and M‐mode images (Figure 1b). Heart rate showed a distinct developmental pattern, increasing sharply from P0 to P8 (*p* < 0.001), followed by a partial decline at P21 and stabilization through Wk8 (Figure 2b, Table 1).

### 3.3 Ejection fraction decreases over postnatal development

LV ejection fraction displayed progressively lower values across developmental timepoints (*p* < 0.001), decreasing from 69 ± 5% at P0 to its lowest value of 60 ± 8% at Week 8 (Figure 2d, Table 1). Fractional shortening (FS) exhibited a similar age‐related decrease in values (*p* = 0.017). Post‐hoc Bland–Altman analysis revealed a systematic bias of −12.3% (95% limits of agreement: +2.9% to −27.4%) between SAX M‐mode and LAX B‐mode derived EF, confirming systematic overestimation by M‐mode across all developmental timepoints (Figure S2).

### 3.4 Diastolic function changes across postnatal development

Transmitral Doppler parameters demonstrated robust age‐related changes. Early filling velocity (E) and late filling velocity (A) both increased significantly with age (*p* < 0.001), with the E/A ratio peaking at P8 and subsequently stabilizing (Table 1). Annular tissue Doppler velocities (e’, a’) and systolic annular velocity (s') increased progressively from P8 to Wk8 (*p* < 0.001 for all). Representative PW Doppler and TDI waveforms illustrating these maturational changes are shown in Figure 1e. Aortic ejection time (AET) remained relatively stable, whereas isovolumic contraction time (IVCT) increased modestly (*p* = 0.02). IVRT trended downward but did not reach statistical significance. LA area, both systolic and diastolic, increased progressively from P8 to Wk8. LA FAC showed significant variation across timepoints (*p* = 0.011) but did not demonstrate a consistent directional trend.

### 3.5 Right ventricular functional maturation

A measure of RV function, tricuspid annular plane systolic excursion (TAPSE) increased progressively from P8 (0.40 ± 0.06 mm) to Week 8 (0.78 ± 0.12 mm) (*p* < 0.001) (Table 1).

### 3.6 Strain measurements reveal selective maturation of myocardial mechanics

Strain analysis showed that most measures of intrinsic myocardial deformation, including global circumferential (MyoGCS, EndoGCS) and radial strain (GRS), remained stable across development (Figure 1c, Table 1). In contrast, myocardial global longitudinal strain (MyoGLS) became significantly more negative, going from −8.79 ± 2.64% at P8 to −11.88 ± 2.38% at P21 (*p* < 0.001).

### 3.7 Sexual dimorphism emerges during late postnatal development

**FIGURE 3:**
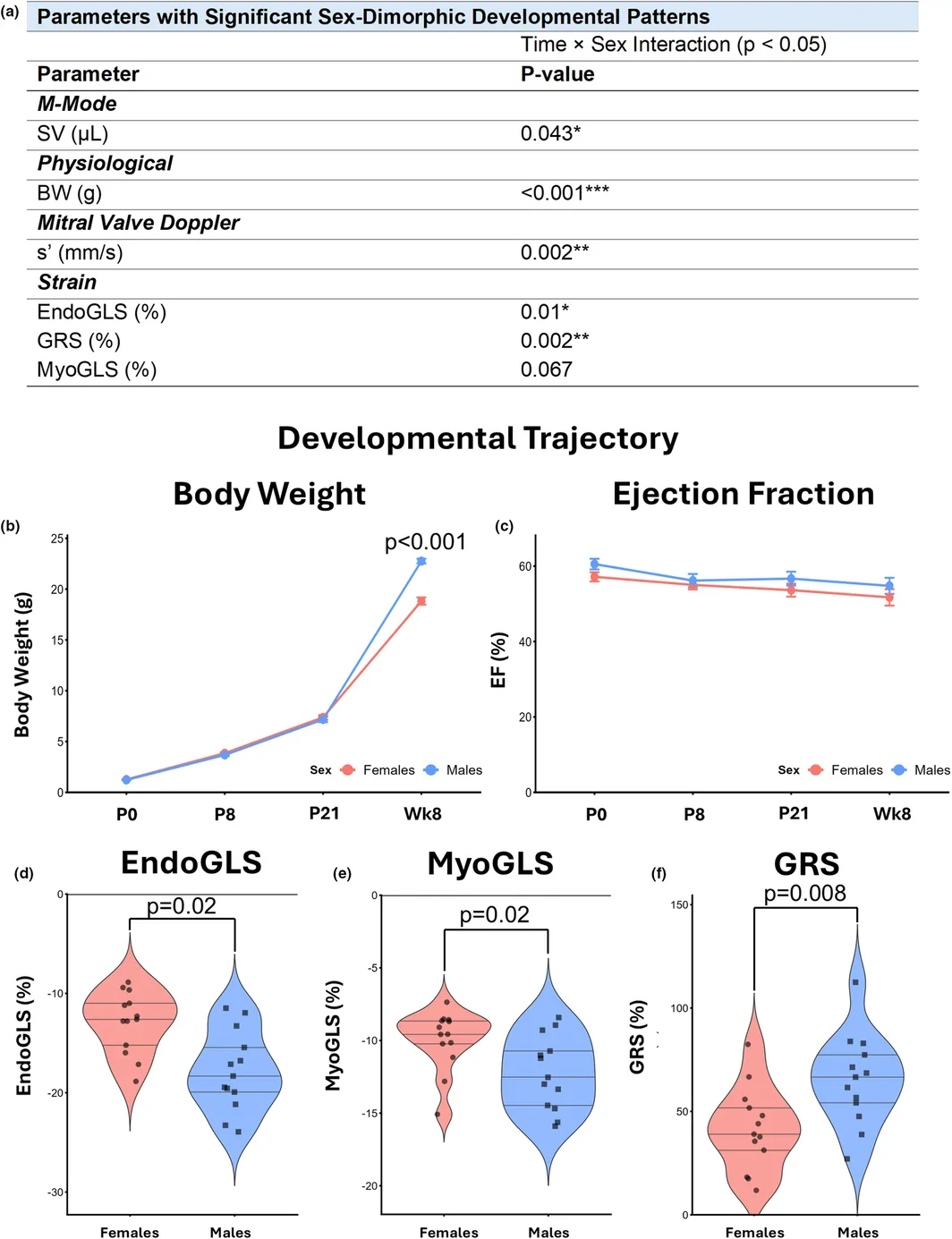
Divergent developmental trajectories of cardiac mechanics in male and female mice. (a) Table of parameters exhibiting significant Time × Sex interaction (*p* < 0.05) identified by two‐way mixed ANOVA. Significance is denoted as follows: *p < 0.05, **p < 0.01, ***p < 0.001. SV, stroke volume; BW, body weight; s', systolic annular velocity; EndoGLS, endocardial global longitudinal strain; GRS, global radial strain; MyoGLS, myocardial global longitudinal strain. (b, c) Developmental trajectories of Heart Rate (HR) and Ejection Fraction (EF) for males (blue) and females (pink) from P8 to Week 8. Data are presented as mean ± SEM. (d–f) Violin plots comparing Wk8 values for: (d) Endocardial Global Longitudinal Strain (EndoGLS), (e) Myocardial Global Longitudinal Strain (MyoGLS), and (f) Global Radial Strain (GRS). *p*‐values derived from independent samples *t*‐tests comparing males versus females at Week 8 time point.

Sex‐stratified analyses revealed several developmental differences (Tables 2 and 3; Tables S1 and S2). Body weight did not differ significantly between males and females until Wk8, at which point males became significantly heavier (*p* < 0.001; Figure 3b). EF did not exhibit a significant Time × Sex interaction (Table S1, Figure 3c).

Most conventional echocardiographic parameters including SAX M‐mode indices, mitral valve Doppler parameters, and TAPSE did not show significant Time × Sex interaction. The exception was stroke volume (SV), which demonstrated a modest but significant Time × Sex interaction (*p* = 0.043; Figure 3a). Beyond stroke volume, the systolic annular velocity (s') also demonstrated a significant Time × Sex interaction (*p* = 0.002; Figure 3a), with males achieving higher peak velocities compared to females by Wk8 (21.52 ± 3.65 vs. 17.33 ± 1.62 mm/s; *p* = 0.002). In contrast, multiple strain parameters exhibited sex‐dimorphic trajectories. Significant Time × Sex interactions were observed for EndoGLS and GRS (*p* < 0.05 for all), whereas MyoGLS exhibited a trend toward a significant interaction (*p* = 0.067). At Wk8, males exhibited more negative longitudinal strain values and higher radial strain compared with females (Figure 3a,d–f, Tables S1 and S2, Figure S1).

## 4 DISCUSSION

This study provides the first prospective myocardial strain‐based echocardiographic characterization of C57BL/6 mice spanning between birth and adulthood. While prior work has assessed early postnatal cardiac development using conventional echocardiographic indices, deformation‐based metrics derived from speckle‐tracking have not previously been defined across this developmental window. Using high‐frequency ultrasound we establish normative, age‐ and sex‐resolved trajectories for advanced functional parameters that are frequently applied in disease models but are often absent from developmental reference datasets (Le & Wagenseil, 2012). These findings enrich the current literature by enabling more accurate interpretation of cardiac phenotypes during neonatal and juvenile cardiac maturation. To facilitate the practical application of these normative values, we also provide a printable echocardiography quicksheet summarizing key developmental parameters across all timepoints (Figure S3).

### 4.1 Baseline murine echocardiography parameters

Consistent with prior work, body weight, LV mass, and LV internal diameter increased progressively with age (Le & Wagenseil, 2012; Vinhas et al., 2013; Zhang et al., 2021). The transient rise in body‐weight–indexed LV mass across the early postnatal and juvenile periods, followed by its decline at adulthood, is consistent with an early phase in which cardiac growth outpaces somatic growth—reflecting the well‐described postnatal transition from cardiomyocyte proliferation to hypertrophic growth—before LV mass and body weight scale proportionally at maturity (Le & Wagenseil, 2012; Zhang et al., 2021). The developmental trajectory of heart rate in our cohort similarly followed a characteristic postnatal pattern, closely mirroring trends previously described using cardiac MRI across neonatal, juvenile, and adult stages in mice (Wiesmann et al., 2000). Although it might seem counterintuitive, the progressively lower values of EF and FS align well with established analyses of cardiac physiology (Wiesmann et al., 2000). While sarcomere force generation increases from birth to adulthood, the disproportionate expansion of LV cavity size relative to stroke volume, coupled with rising systemic afterload, results in lower volumetric indices of systolic function. Importantly, strain measures remained stable, providing further support to the notion that intrinsic contractility is preserved despite geometric remodeling (Le & Wagenseil, 2012; Siedner et al., 2003; Wiesmann et al., 2000). The stability of these strain measures across postnatal development has important implications beyond establishing normative values, as myocardial strain has emerged as a particularly sensitive tool for detecting early cardiac dysfunction in preclinical disease models (Bauer et al., 2011; de Lucia et al., 2019; Hoffman et al., 2019; Zheng et al., 2017). Prior work has demonstrated that strain detects subclinical functional impairment earlier than conventional echocardiography in models of dilated cardiomyopathy (Zheng et al., 2017), aging (de Lucia et al., 2019), and sepsis (Hoffman et al., 2019), underscoring its growing utility in early‐stage phenotyping and mechanistic studies.

Myocardial strain quantifies deformation of the ventricular wall during the cardiac cycle and, at the tissue level, integrates the shortening and thickening of individual cardiomyocytes and their constituent sarcomeres. Longitudinal and circumferential strain reflect myocyte shortening along the base‐to‐apex and short‐axis circumferential directions, respectively, whereas radial strain reflects thickening of the wall toward the ventricular cavity; more negative longitudinal and circumferential values, and more positive radial values, indicate greater deformation and, in general, more vigorous contraction. Because these indices depend on the coordinated behavior of contractile units rather than on chamber geometry, a decline in their magnitude typically signals impaired intrinsic contractility and often precedes changes in load‐dependent indices such as ejection fraction. At the subcellular level, high‐resolution in vivo nano‐imaging of the beating mouse heart has demonstrated that the dynamics and synchrony of sarcomere‐length changes—rather than absolute sarcomere length—govern ventricular pump function (Kobirumaki‐Shimozawa et al., 2016; Kobirumaki‐Shimozawa et al., 2021). Within this framework, the stability of global circumferential and radial strain that we observed across postnatal development suggests that, despite pronounced somatic and chamber growth, the coordinated sarcomere‐level deformation underlying pump function is established early and preserved through maturation, in parallel with the known maturation of calcium‐handling and sarcomeric proteins during this period (Guo & Pu, 2020; Siedner et al., 2003).

Diastolic parameters, including progressive increases in E, A, and E/A ratio, mirror the previously reported developmental trends in these parameters that eventually plateau by adulthood (Le & Wagenseil, 2012; Vinhas et al., 2013). Furthermore, the heart rate stabilization observed from P21 onward aligns with studies comparing young and old mice (Figure 2b) (Zhang et al., 2021). Comprehensive diastolic normative data for adult rodents have been standardized across multiple laboratories (Zacchigna et al., 2021), providing an important complement to the developmental trajectories reported here.

These echocardiographic trends reflect the profound physiological transition occurring during the postnatal period (Sakamoto & Kelly, 2024). Shortly after birth, cardiomyocytes shift from a glycolytic metabolic profile to fatty‐acid oxidation (FAO), a process that increases mitochondrial density and oxidative capacity (Dorn 2nd et al., 2015). This metabolic maturation likely contributes to the observed stabilization of strain measures despite rapid growth. Concurrently, calcium‐handling machinery is known to undergo significant refinement, including increased expression of SERCA2a and maturation of RyR2‐mediated calcium release (Liu et al., 2002), processes that likely contribute to the enhanced excitation–contraction coupling efficiency (Guo & Pu, 2020). Finally, the well‐documented cessation of cardiomyocyte proliferation and the onset of hypertrophic growth are consistent with the progressive expansion of LV mass and the accompanying changes in load‐dependent indices such as EF observed here.

### 4.2 Sex‐dependent differences

We identified distinct sex‐dependent developmental trajectories, characterized by significant Time × Sex interactions for body weight, strain parameters, and stroke volume (Figure 3a). The most pronounced difference occurred at Wk8, where males exhibited greater longitudinal and radial strain magnitudes than females. Although ejection fraction values were generally higher in males (Figure 3c), this difference did not reach a significant Time × Sex interaction. These findings suggest that strain imaging is highly sensitive to subtle functional changes that are not detected by conventional functional indices. The emergence of sex differences by P21 further highlights this time point as a critical transitional period in postnatal cardiac maturation, though further mechanistic studies are needed to define the underlying biological drivers. Similarly, systolic annular velocity (s'), a tissue Doppler index of longitudinal systolic function, demonstrated a significant Time × Sex interaction (*p* = 0.002), with males exhibiting higher peak velocities at Wk8. This finding extends the sex‐dimorphic pattern beyond speckle‐tracking strain to include tissue Doppler‐derived indices, suggesting that the emergence of greater male contractility at adulthood is detectable across multiple echocardiographic modalities. Our findings are supported by prior in vitro work showing enhanced contractility and relaxation in isolated cardiomyocytes from adult male mice compared to ones isolated from female mice (Emerson et al., 2023). Interestingly, this pattern reverses later in adulthood, where females often outperform males in certain measures of cardiac function (Koch et al., 2013), suggesting dynamic sex‐dependent shifts across the lifespan.

The greater longitudinal and radial strain magnitudes observed in males by Wk8 are consistent with well‐documented sex differences in cardiomyocyte contractile and calcium‐handling properties. Isolated male cardiomyocytes generally display greater contractility than female ones, and these differences are shaped by sex‐steroid signaling: testosterone modulates SERCA‐dependent Ca^2+^ handling and myofilament function, while female cardiomyocytes exhibit distinct ryanodine‐receptor organization and Ca^2+^‐release characteristics (Ayaz & Howlett, 2015; Emerson et al., 2023; Laasmaa et al., 2023; Parks & Howlett, 2013). Notably, the emergence of these strain differences specifically between P21 and Wk8 coincides with pubertal maturation and the associated rise in circulating androgens, offering a plausible hormonal basis for the late‐adolescent divergence we observed.

The sex‐dependent strain patterns we observed here diverge from human myocardial mechanics. In clinical populations, females typically exhibit more negative strain values, indicating greater myocardial deformation compared with males (Dalen et al., 2010; Heydari et al., 2023; Pillai et al., 2025). Several biological factors may account for this discrepancy. First, fundamental species differences in myosin heavy‐chain (MHC) isoform expression are a likely contributing factor, as adult murine ventricles predominantly express α‐MHC (fast kinetics), whereas human ventricles express 𝛽‐MHC (slow kinetics) (Lu et al., 2022; Reiser et al., 2001). Second, the 10‐fold difference in heart rate between species fundamentally alters the contraction‐relaxation dynamics governing strain. Finally, sex differences in cardiac function are dynamic and evolve with age. Although male mice in our study exhibited greater myocardial strain at 8 weeks, aging studies suggest that this relationship may reverse later in life, with females maintaining better contractility at older ages (Koch et al., 2013; Le & Wagenseil, 2012; Wiesmann et al., 2000). Together, these species‐level differences underscore the importance of using species‐specific normative values when interpreting sex‐dependent strain patterns.

### 4.3 Technical considerations and common pitfalls

High‐quality echocardiographic data in mice requires careful attention to acquisition techniques and analysis, particularly across developmental timepoints where animal size, heart rate, and anatomy change substantially. The following considerations reflect practical lessons intended to help guide investigators applying these methods in their own laboratories to increase reproducibility and decrease variability. To minimize observer bias, we strongly recommend that image acquisition and offline analysis be performed by investigators who are blinded to group assignment and experimental conditions, particularly given that unconscious bias can influence measurement decisions such as M‐mode cursor placement in developmental and disease model studies (Kilkenny et al., 2010). A post‐hoc Bland–Altman analysis performed in our cohort confirmed a systematic bias of −12.3% (95% limits of agreement: +2.9% to −27.4%) between SAX M‐mode and LAX B‐mode derived EF values (Figure S2), consistent with prior reports (Kacira et al., 2024; Kacira et al., 2025), and investigators are encouraged to use B‐mode derived volumetric assessment when studying disease models with regional wall motion abnormalities.

One of the most critical aspects is maintaining normothermia, particularly in neonatal animals. Neonatal mice are unable to thermoregulate independently and are highly susceptible to hypothermia‐induced bradycardia, which will confound functional measurements. The ultrasound gel warmer should be turned on at least 30 min prior to imaging, and in addition to the heated platform, a supplemental heat lamp should be available. Furthermore, ECG electrode contact is a practical challenge in neonatal mice, whose limbs do not reach the platform electrodes. Applying electrode gel with a syringe to draw a conductive line between the animal's limbs and the electrodes resolves this reliably and allows continuous heart rate monitoring. When handling neonatal pups, rubbing gloved hands in the home cage bedding and nesting material before and after each session transfers familiar scent and minimizes the risk of maternal rejection upon return to the litter. This precaution is particularly important at P0 and P8, when pups are most vulnerable, and represents a critical step for maintaining litter integrity in longitudinal neonatal studies.

A common source of SAX M‐mode measurement error is incorrect placement of the sample volume. The sample volume should be positioned at the level of maximal LV diameter, perpendicular to the interventricular septum, at the mid‐papillary level (See Appendix S1). Inclusion of papillary muscle within the sample volume is a frequent pitfall that falsely elevates posterior wall thickness and leads to overestimation of EF. The papillary muscles should always be visible in the 2D B‐mode guide image but must not overlap with the M‐mode cursor. During analysis, all measurements should be made between breaths to minimize respiratory motion artifacts, and a minimum of two to three cardiac cycles should be averaged per measurement. Additionally, SAX M‐mode derived volumetric parameters (LVEDV, LVESV, EF, SV, CO) rely on the Teichholz formula, which assumes a spherical LV geometry. While reproducible and appropriate for healthy developmental normative values, this geometric assumption may overestimate true volumes in remodeled or diseased models, especially when there are regional changes in the wall (e.g., myocardial infarction models) (Kacira et al., 2025). During mitral valve Doppler acquisition, rib cage and sternum shadowing is a common artifact that prevents clean mitral inflow Doppler signals. In this case, minor adjustments in the *Y*‐ or *X*‐axis imaging plane, slight transducer rotation or a 5–10 degree platform tilt to find a better acoustic window between ribs, will often resolve the issue. Importantly, the Doppler angle relative to the direction of blood flow should not exceed 45 degrees, as larger angles systematically underestimate true velocities. For speckle‐tracking strain analysis, high quality LAX B‐mode cine loops are important. Investigators should select cine loops with the clearest possible endocardial border definition and exclude frames affected by respiratory motion before initiating tracking. The tracking should be carefully reviewed after automated processing and manual correction of the trace should be applied as needed. For the LA area measurements, we used the parasternal long‐axis view rather than the apical four‐chamber view, as we found this approach more reliable and reproducible across developmental timepoints. Maximum LA area (systole) corresponds to the end of the T wave on the ECG, and minimum LA area (diastole) corresponds to the QRS complex. Excessive transducer pressure during apical four‐chamber view acquisition may compress the atria leading to underestimation of LA dimensions. Adherence to these acquisition and analysis standards is essential for generating reproducible measurements and for appropriate application of the normative values provided in this study.

### 4.4 Limitations and future directions

Our findings must be interpreted in the context of several limitations. First, anesthesia significantly influences cardiac hemodynamics (Roth et al., 2002; Tuohy et al., 2025). However, we minimized variability by using a standardized isoflurane protocol to avoid the stress‐induced tachycardia and motion artifacts associated with conscious imaging (Gao et al., 2011; Lindsey et al., 2018; Popp et al., 2025; Roth et al., 2002). Secondly, the data presented here were obtained exclusively in C57BL/6 mice from Jackson Laboratories, and cardiac developmental trajectories may differ across strains or substrains (Garcia‐Menendez et al., 2013). Third, all volumetric parameters (LVEDV, LVESV, EF, SV) were derived from SAX M‐mode using the Teichholz formula, which assumes symmetric LV geometry and systematically overestimates EF compared to B‐mode derived Simpson's monoplane estimates (bias −12.3%, 95% limits of agreement +2.9% to −27.4%; see Technical Considerations). This limitation does not affect strain measurements, which quantify myocardial deformation through speckle‐tracking and do not rely on geometric assumptions. Finally, echocardiographic image quality can vary with animal size, particularly in neonates, as well as with differences in instrumentation and operator skill, though we mitigated this by using a high‐resolution platform and standardized acquisition. As a result of these neonatal imaging constraints, speckle‐tracking strain analysis, mitral valve doppler, LA area and RV assessment were not performed at P0, representing a gap in the earliest developmental window characterized here. From P8 onward, all measurements were successfully obtained in all animals with no exclusions.

While 2D speckle‐tracking echocardiography offers high temporal resolution and is widely accessible on standard small‐animal ultrasound systems, it is inherently limited by through‐plane motion and the need to assume ventricular geometry, both of which can introduce tracking error or measurement variability (Damen et al., 2021). In contrast, four‐dimensional ultrasound (4DUS) overcomes these constraints by acquiring volumetric datasets throughout the cardiac cycle, reducing geometric assumptions and improving reproducibility for volumetric and regional functional measurements (Rutledge et al., 2020). 4DUS has shown advantages in models with regional wall‐motion abnormalities, like myocardial infarction, where asymmetric remodeling limits the accuracy of 2D‐derived indices (Dann et al., 2022). However, 4DUS requires specialized hardware, longer acquisition times, and more complex post‐processing workflows. For these reasons, 2D speckle‐tracking remains the more practical and widely adopted modality (Rutledge et al., 2020).

In the current study, RV function was evaluated using TAPSE as a surrogate of longitudinal RV systolic function; however, a truly comprehensive RV characterization would require additional parameters including RV fractional area change, RV free wall thickness, and tricuspid inflow Doppler indices, none of which were systematically acquired in this cohort. Prior studies have characterized RV functional parameters in murine models of pulmonary hypertension and right heart failure, providing complementary reference frameworks for investigators focused on right‐sided cardiac physiology (Cheng et al., 2014; Spyropoulos et al., 2020; Spyropoulos et al., 2021; Zhu et al., 2019). Furthermore, large vessel assessment, including aortic and pulmonary artery dimensions, flow velocities, and indices of vascular stiffness, was not performed, representing an additional gap in the characterization of the developing cardiovascular system. Future work should aim to expand these normative values by incorporating comprehensive right ventricular assessment, as well as large vessel characterization across postnatal development. Additionally, integrating non‐invasive imaging with invasive pressure‐volume loop analysis would provide gold‐standard validation of these developmental metrics, although such methods are limited by their terminal nature and increased technical difficulty in neonatal mice. Finally, extending these serial observations into adulthood and onward to senescence would further contextualize how early‐life sex differences evolve across the lifespan.

## AUTHOR CONTRIBUTIONS

**Sehjin Jo:** Data curation; formal analysis; investigation. **George Spyropoulos:** Data curation; formal analysis. **Logan G. Hart:** Data curation; formal analysis; investigation. **Taylor A. Covington:** Data curation; formal analysis; investigation. **Arvind K. Pandey:** Formal analysis; investigation. **Thomas Michel:** Funding acquisition; resources. **Fotios Spyropoulos:** Conceptualization; data curation; formal analysis; funding acquisition; investigation; methodology; supervision; visualization.

## FUNDING INFORMATION

This study was supported by National Institutes of Health (NIH) Grants K08HL168240 to F.S.; NIH grants R33HL157918, R21AG063073 and R01HL152173 to T.M.

## CONFLICT OF INTEREST STATEMENT

The authors declare no conflicts of interest.

## ETHICS STATEMENT

All animal procedures were reviewed and approved by the Institutional Animal Care and Use Committee (IACUC) of Brigham and Women’s Hospital, under the oversight of the BWH Center for Comparative Medicine (protocol 2016N000278). All experiments were performed in accordance with the National Institutes of Health Guide for the Care and Use of Laboratory Animals.

## Supporting information


**Appendix S1.** Protocol for Image acquisition and data analysis.

**Figure S1.** Longitudinal sex differences in strain. (a, b) Line graphs illustrating developmental trajectories for (a) Endocardial global longitudinal strain (EndoGLS) and (b) Global radial strain (GRS) in male (blue) and female (pink) mice from P8 to Wk8. Data are presented as mean ± SEM. *p*‐values derived from independent samples *t*‐tests comparing males versus females at each time point. Significance: **p < 0.01.
**Figure S2.** Bland–altman analysis comparing M‐mode and B‐mode derived ejection fraction across postnatal development. The difference between B‐mode (Simpson's monoplane) and M‐mode (Teichholz formula) derived ejection fraction (EF) is plotted against the mean of the two methods for each animal at each developmental timepoint. The solid black horizontal line represents the mean bias (−12.3%), indicating that M‐mode systematically overestimates EF relative to B‐mode across all timepoints. Red dashed lines indicate the 95% limits of agreement (ULoA: +2.9%; LLoA: −27.4%). Data points are color‐ and shape‐coded by developmental timepoint: P0 (orange circles), P8 (green squares), P21 (blue triangles), and Week 8 (pink diamonds).
**Figure S3:** Echocardiography reference quicksheet. Summary of key reference values derived from Table 1, formatted for use as a bench‐side reference during image acquisition and analysis.
**Table S1.** Statistical analysis of sex‐specific differences in postnatal cardiac development.
**Table S2.** Sex‐specific comparisons at each developmental time point.
**Table S3.** Echocardiographic measurement feasibility.

Movie S1.

Movie S2.

Movie S3.

Movie S4.

Movie S5.

Movie S6.

Movie S7.

Movie S8.

Movie S9.

## Data Availability

Data will be made available upon reasonable request.

## References

[phy271113-bib-0001] Ayaz, O. , & Howlett, S. E. (2015). Testosterone modulates cardiac contraction and calcium homeostasis: Cellular and molecular mechanisms. Biology of Sex Differences, 6, 9. 10.1186/s13293-015-0027-9 PMC4411792 25922656

[phy271113-bib-0002] Barros Filho, A. C. L. , Moreira, H. T. , Dias, B. P. , Ribeiro, F. F. F. , Tanaka, D. M. , Schmidt, A. , Maciel, B. C. , Simões, M. V. , Marin‐Neto, J. A. , & Romano, M. M. D. (2021). Feasibility and reference intervals assessed by conventional and speckle‐tracking echocardiography in normal hamsters. Physiological Reports, 9(5), e14776. 10.14814/phy2.14776 PMC7923569 33650789

[phy271113-bib-0003] Bauer, M. , Cheng, S. , Jain, M. , Ngoy, S. , Theodoropoulos, C. , Trujillo, A. , Lin, F. C. , & Liao, R. (2011). Echocardiographic speckle‐tracking based strain imaging for rapid cardiovascular phenotyping in mice. Circulation Research, 108(8), 908–916. 10.1161/CIRCRESAHA.110.239574 PMC3376717 21372284

[phy271113-bib-0004] Cheng, H. W. , Fisch, S. , Cheng, S. , Bauer, M. , Ngoy, S. , & Qiu, Y. (2014). Assessment of right ventricular structure and function in mouse model of pulmonary artery constriction by transthoracic echocardiography. Journal of Visualized Experiments: JoVE, 84, e51041. 10.3791/51041 PMC4397999 24513696

[phy271113-bib-0005] Dalen, H. , Thorstensen, A. , Aase, S. A. , Ingul, C. B. , Torp, H. , Vatten, L. J. , & Stoylen, A. (2010). Segmental and global longitudinal strain and strain rate based on echocardiography of 1266 healthy individuals: The HUNT study in Norway. European Journal of Echocardiography, 11(2), 176–183. 10.1093/ejechocard/jep194 19946115

[phy271113-bib-0006] Damen, F. W. , Salvas, J. P. , Pereyra, A. S. , Ellis, J. M. , & Goergen, C. J. (2021). Improving characterization of hypertrophy‐induced murine cardiac dysfunction using four‐dimensional ultrasound‐derived strain mapping. American Journal of Physiology. Heart and Circulatory Physiology, 321(1), H197–H207. 10.1152/ajpheart.00133.2021 PMC8321816 34085843

[phy271113-bib-0007] Dann, M. M. , Clark, S. Q. , Trzaskalski, N. A. , Earl, C. C. , Schepers, L. E. , Pulente, S. M. , Lennord, E. N. , Annamalai, K. , Gruber, J. M. , Cox, A. D. , Lorenzen‐Schmidt, I. , Seymour, R. , Kim, K. H. , Goergen, C. J. , & Mulvihill, E. E. (2022). Quantification of murine myocardial infarct size using 2‐D and 4‐D high‐frequency ultrasound. American Journal of Physiology. Heart and Circulatory Physiology, 322(3), H359–H372. 10.1152/ajpheart.00476.2021 PMC8836752 34995167

[phy271113-bib-0008] de Lucia, C. , Wallner, M. , Eaton, D. M. , Zhao, H. , Houser, S. R. , & Koch, W. J. (2019). Echocardiographic strain analysis for the early detection of left ventricular systolic/diastolic dysfunction and dyssynchrony in a mouse model of physiological aging. The Journals of Gerontology. Series A, Biological Sciences and Medical Sciences, 74(4), 455–461. 10.1093/gerona/gly139 PMC6417453 29917053

[phy271113-bib-0009] Dorn, G. W., 2nd , Vega, R. B. , & Kelly, D. P. (2015). Mitochondrial biogenesis and dynamics in the developing and diseased heart. Genes & Development, 29(19), 1981–1991. 10.1101/gad.269894.115 PMC4604339 26443844

[phy271113-bib-0010] Emerson, J. I. , Ariel, P. , Shi, W. , & Conlon, F. L. (2023). Sex differences in mouse cardiac electrophysiology revealed by simultaneous imaging of excitation‐contraction coupling. Journal of Cardiovascular Development and Disease, 10(12), 479. 10.3390/jcdd10120479 PMC10743987 38132647

[phy271113-bib-0011] Gao, S. , Ho, D. , Vatner, D. E. , & Vatner, S. F. (2011). Echocardiography in mice. Current Protocols Mouse Biology, 1, 71–83. 10.1002/9780470942390.mo100130 PMC3130310 21743841

[phy271113-bib-0012] Garcia‐Menendez, L. , Karamanlidis, G. , Kolwicz, S. , & Tian, R. (2013). Substrain specific response to cardiac pressure overload in C57BL/6 mice. American Journal of Physiology. Heart and Circulatory Physiology, 305(3), H397–H402. 10.1152/ajpheart.00088.2013 PMC3742875 23709599

[phy271113-bib-0013] Guo, Y. , & Pu, W. T. (2020). Cardiomyocyte maturation: New phase in development. Circulation Research, 126(8), 1086–1106. 10.1161/CIRCRESAHA.119.315862 PMC7199445 32271675

[phy271113-bib-0014] Heydari, B. , Satriano, A. , Jerosch‐Herold, M. , Kolm, P. , Kim, D. Y. , Cheng, K. , Choi, Y. L. , Antiochos, P. , White, J. A. , Mahmod, M. , Chan, K. , Raman, B. , Desai, M. Y. , Ho, C. Y. , Dolman, S. F. , Desvigne‐Nickens, P. , Maron, M. S. , Friedrich, M. G. , Schulz‐Menger, J. , … HCMR Investigators . (2023). 3‐dimensional strain analysis of hypertrophic cardiomyopathy: Insights from the NHLBI international HCM registry. JACC: Cardiovascular Imaging, 16(4), 478–491. 10.1016/j.jcmg.2022.10.005 PMC10802851 36648040

[phy271113-bib-0015] Hoffman, M. , Kyriazis, I. D. , Lucchese, A. M. , de Lucia, C. , Piedepalumbo, M. , Bauer, M. , Schulze, P. C. , Bonios, M. J. , Koch, W. J. , & Drosatos, K. (2019). Myocardial strain and cardiac output are preferable measurements for cardiac dysfunction and can predict mortality in septic mice. Journal of the American Heart Association, 8(10), e012260. 10.1161/JAHA.119.012260 PMC6585345 31112430

[phy271113-bib-0016] Kacira, E. , Oueis, M. F. , Tamimi, N. H. , Sturgill, S. L. , Xu, X. , Hund, T. J. , Ziolo, M. T. , Han, Y. , Deschênes, I. , & Fu, J. D. (2025). Advanced 4‐chamber echocardiography techniques enable clinically matched precise characterization of heart disease progression in mice. Commun Med (Lond), 5(1), 325. 10.1038/s43856-025-01036-w PMC12314085 40745225

[phy271113-bib-0017] Kacira, E. , Pittman, M. , Yang, D. , Sturgill, S. L. , Ziolo, M. T. , Han, Y. , Deschênes, I. , & Fu, J. D. (2024). Long‐Axis biplane echocardiography sensitively detects the progressing functional deterioration of mouse heart. Circulation. Heart Failure, 17(1), e011046. 10.1161/CIRCHEARTFAILURE.123.011046 PMC10872990 38054280

[phy271113-bib-0018] Kilkenny, C. , Browne, W. J. , Cuthill, I. C. , Emerson, M. , & Altman, D. G. (2010). Improving bioscience research reporting: The ARRIVE guidelines for reporting animal research. PLoS Biology, 8(6), e1000412. 10.1371/journal.pbio.1000412 PMC2893951 20613859

[phy271113-bib-0019] Kirsanov, O. , & Geyer, C. B. (2023). Pharmacological treatment of neonatal and juvenile mice to study spermatogenesis. Methods in Molecular Biology, 2656, 227–237. 10.1007/978-1-0716-3139-3_13 PMC10473193 37249875

[phy271113-bib-0020] Kobirumaki‐Shimozawa, F. , Oyama, K. , Shimozawa, T. , Mizuno, A. , Ohki, T. , Terui, T. , Minamisawa, S. , Ishiwata, S.’. , & Fukuda, N. (2016). Nano‐imaging of the beating mouse heart in vivo: Importance of sarcomere dynamics, as opposed to sarcomere length per se, in the regulation of cardiac function. The Journal of General Physiology, 147(1), 53–62. 10.1085/jgp.201511484 PMC4692490 26712849

[phy271113-bib-0021] Kobirumaki‐Shimozawa, F. , Shimozawa, T. , Oyama, K. , Baba, S. , Li, J. , Nakanishi, T. , Terui, T. , Louch, W. E. , Ishiwata, S.’. , & Fukuda, N. (2021). Synchrony of sarcomeric movement regulates left ventricular pump function in the in vivo beating mouse heart. The Journal of General Physiology, 153(11), e202012860. 10.1085/jgp.202012860 PMC8493835 34605861

[phy271113-bib-0022] Koch, S. E. , Haworth, K. J. , Robbins, N. , Smith, M. A. , Lather, N. , Anjak, A. , Jiang, M. , Varma, P. , Jones, W. K. , & Rubinstein, J. (2013). Age‐ and gender‐related changes in ventricular performance in wild‐type FVB/N mice as evaluated by conventional and vector velocity echocardiography imaging: A retrospective study. Ultrasound in Medicine & Biology, 39(11), 2034–2043. 10.1016/j.ultrasmedbio.2013.04.002 PMC4857602 23791351

[phy271113-bib-0023] Krishnan, A. , Samtani, R. , Dhanantwari, P. , Lee, E. , Yamada, S. , Shiota, K. , Donofrio, M. T. , Leatherbury, L. , & Lo, C. W. (2014). A detailed comparison of mouse and human cardiac development. Pediatric Research, 76(6), 500–507. 10.1038/pr.2014.128 PMC4233008 25167202

[phy271113-bib-0024] Laasmaa, M. , Branovets, J. , Stolova, J. , Shen, X. , Ratsepso, T. , & Balodis, M. J. (2023). Cardiomyocytes from female compared to male mice have larger ryanodine receptor clusters and higher calcium spark frequency. The Journal of Physiology, 601(18), 4033–4052. 10.1113/JP284515 37561554

[phy271113-bib-0025] Le, V. P. , & Wagenseil, J. E. (2012). Echocardiographic characterization of postnatal development in mice with reduced arterial elasticity. Cardiovascular Engineering and Technology, 3(4), 424–438. 10.1007/s13239-012-0108-4 PMC3640570 23646094

[phy271113-bib-0026] Lindsey, M. L. , Kassiri, Z. , Virag, J. A. I. , de Castro Bras, L. E. , & Scherrer‐Crosbie, M. (2018). Guidelines for measuring cardiac physiology in mice. American Journal of Physiology. Heart and Circulatory Physiology, 314(4), H733–H752. 10.1152/ajpheart.00339.2017 PMC5966769 29351456

[phy271113-bib-0027] Liu, W. , Yasui, K. , Opthof, T. , Ishiki, R. , Lee, J. K. , Kamiya, K. , Yokota, M. , & Kodama, I. (2002). Developmental changes of Ca(2+) handling in mouse ventricular cells from early embryo to adulthood. Life Sciences, 71(11), 1279–1292. 10.1016/s0024-3205(02)01826-x 12106593

[phy271113-bib-0028] Lu, P. , Wu, B. , Feng, X. , Cheng, W. , Kitsis, R. N. , & Zhou, B. (2022). Cardiac myosin heavy chain reporter mice to study heart development and disease. Circulation Research, 131(4), 364–366. 10.1161/CIRCRESAHA.122.321461 PMC9357176 35862119

[phy271113-bib-0029] Parks, R. J. , & Howlett, S. E. (2013). Sex differences in mechanisms of cardiac excitation‐contraction coupling. Pflügers Archiv, 465(5), 747–763. 10.1007/s00424-013-1233-0 PMC3651827 23417603

[phy271113-bib-0030] Pillai, R. , Zhang, L. , Peters, K. , Jha, V. , O'Donnell, C. J. , Manning, W. J. , & Tsao, C. W. (2025). Age‐ and sex‐differences and reference values for ventricular strain by cardiovascular magnetic resonance imaging in adults without cardiovascular disease or cardiovascular disease risk factors. Journal of Cardiovascular Magnetic Resonance, 27(1), 101902. 10.1016/j.jocmr.2025.101902 PMC12138549 40300666

[phy271113-bib-0031] Popp, J. L. , Patel, K. , Cuomo, D. , Lynch, R. M. , Leung, S. W. , Berridge, B. R. , Rusyn, I. , Chiu, W. A. , & Threadgill, D. W. (2025). Derivation of ultrasound and electrocardiogram cardiac reference ranges for Mus musculus using the collaborative cross and identification of new cardiac models. BMC Cardiovascular Disorders, 25(1), 684. 10.1186/s12872-025-05156-y PMC12465196 41013247

[phy271113-bib-0032] Reiser, P. J. , Portman, M. A. , Ning, X. H. , & Schomisch Moravec, C. (2001). Human cardiac myosin heavy chain isoforms in fetal and failing adult atria and ventricles. American Journal of Physiology. Heart and Circulatory Physiology, 280(4), H1814–H1820. 10.1152/ajpheart.2001.280.4.H1814 11247796

[phy271113-bib-0033] Roth, D. M. , Swaney, J. S. , Dalton, N. D. , Gilpin, E. A. , & Ross, J., Jr. (2002). Impact of anesthesia on cardiac function during echocardiography in mice. American Journal of Physiology. Heart and Circulatory Physiology, 282(6), H2134–H2140. 10.1152/ajpheart.00845.2001 12003821

[phy271113-bib-0034] Rutledge, C. , Cater, G. , McMahon, B. , Guo, L. , Nouraie, S. M. , Wu, Y. , Villanueva, F. , & Kaufman, B. A. (2020). Commercial 4‐dimensional echocardiography for murine heart volumetric evaluation after myocardial infarction. Cardiovascular Ultrasound, 18(1), 9. 10.1186/s12947-020-00191-5 PMC7068892 32164714

[phy271113-bib-0035] Sakamoto, T. , & Kelly, D. P. (2024). Cardiac maturation. Journal of Molecular and Cellular Cardiology, 187, 38–50. 10.1016/j.yjmcc.2023.12.008 PMC10923079 38160640

[phy271113-bib-0036] Siedner, S. , Kruger, M. , Schroeter, M. , Metzler, D. , Roell, W. , Fleischmann, B. K. , Hescheler, J. , Pfitzer, G. , & Stehle, R. (2003). Developmental changes in contractility and sarcomeric proteins from the early embryonic to the adult stage in the mouse heart. The Journal of Physiology, 548(Pt 2), 493–505. 10.1113/jphysiol.2002.036509 PMC2342849 12640016

[phy271113-bib-0037] Spyropoulos, F. , Das, A. A. , Waldeck‐Weiermair, M. , Yadav, S. , Pandey, A. K. , & Guo, R. (2024). Adult and neonatal models of chemogenetic heart failure caused by oxidative stress. The Journal of Clinical Investigation, 134, e178251. 10.1172/JCI178251 PMC11060721 38483521

[phy271113-bib-0038] Spyropoulos, F. , Michael, Z. , Finander, B. , Vitali, S. , Kosmas, K. , Zymaris, P. , Kalish, B. T. , Kourembanas, S. , & Christou, H. (2021). Acetazolamide improves right ventricular function and metabolic gene dysregulation in experimental pulmonary arterial hypertension. Frontiers in Cardiovascular Medicine, 8, 662870. 10.3389/fcvm.2021.662870 PMC8247952 34222363

[phy271113-bib-0039] Spyropoulos, F. , Vitali, S. H. , Touma, M. , Rose, C. D. , Petty, C. R. , Levy, P. , Kourembanas, S. , & Christou, H. (2020). Echocardiographic markers of pulmonary hemodynamics and right ventricular hypertrophy in rat models of pulmonary hypertension. Pulmonary Circuit, 10(2), 2045894020910976. 10.1177/2045894020910976 PMC7268140 32537128

[phy271113-bib-0040] Tuohy, K. P. , Tynan, A. R. , Gerisma, J. N. , & Marx, J. O. (2025). Effect of mouse (Mus musculus) sex and C57BL/6 substrain on sensitivity to isoflurane and ketamine‐xylazine‐acepromazine anesthesia. Journal of the American Association for Laboratory Animal Science, 64(4), 1–10. 10.30802/AALAS-JAALAS-25-038 PMC12530266 40683645

[phy271113-bib-0041] Vinhas, M. , Araujo, A. C. , Ribeiro, S. , Rosario, L. B. , & Belo, J. A. (2013). Transthoracic echocardiography reference values in juvenile and adult 129/Sv mice. Cardiovascular Ultrasound, 11, 12. 10.1186/1476-7120-11-12 PMC3651272 23634975

[phy271113-bib-0042] Wiesmann, F. , Ruff, J. , Hiller, K. H. , Rommel, E. , Haase, A. , & Neubauer, S. (2000). Developmental changes of cardiac function and mass assessed with MRI in neonatal, juvenile, and adult mice. American Journal of Physiology. Heart and Circulatory Physiology, 278(2), H652–H657. 10.1152/ajpheart.2000.278.2.H652 10666098

[phy271113-bib-0043] Yu, Q. , Leatherbury, L. , Tian, X. , & Lo, C. W. (2008). Cardiovascular assessment of fetal mice by in utero echocardiography. Ultrasound in Medicine & Biology, 34(5), 741–752. 10.1016/j.ultrasmedbio.2007.11.001 PMC4275222 18328616

[phy271113-bib-0044] Zacchigna, S. , Paldino, A. , Falcao‐Pires, I. , Daskalopoulos, E. P. , Dal Ferro, M. , & Vodret, S. (2021). Towards standardization of echocardiography for the evaluation of left ventricular function in adult rodents: A position paper of the ESC working group on myocardial function. Cardiovascular Research, 117(1), 43–59. 10.1093/cvr/cvaa110 32365197

[phy271113-bib-0045] Zhang, T. Y. , Zhao, B. J. , Wang, T. , & Wang, J. (2021). Effect of aging and sex on cardiovascular structure and function in wildtype mice assessed with echocardiography. Scientific Reports, 11(1), 22800. 10.1038/s41598-021-02196-0 PMC8611093 34815485

[phy271113-bib-0046] Zheng, M. , Pan, F. , Liu, Y. , Li, Z. , Zhou, X. , Meng, X. , Liu, L. , & Ge, S. (2017). Echocardiographic strain analysis for the early detection of myocardial structural abnormality and initiation of drug therapy in a mouse model of dilated cardiomyopathy. Ultrasound in Medicine & Biology, 43(12), 2914–2924. 10.1016/j.ultrasmedbio.2017.07.020 28942269

[phy271113-bib-0047] Zhu, Z. , Godana, D. , Li, A. , Rodriguez, B. , Gu, C. , Tang, H. , Minshall, R. D. , Huang, W. , & Chen, J. (2019). Echocardiographic assessment of right ventricular function in experimental pulmonary hypertension. Pulmonary Circulation, 9(2), 2045894019841987. 10.1177/2045894019841987 PMC6566495 30942120

