# Supplementary material for: Prospective echocardiographic characterization of postnatal cardiac development in C57BL/6 mice: Strain analysis between birth and adulthood
Source: Physiol Rep. 2026 Sep 24;14(18):e71113. doi: 10.14814/phy2.71113 (PMC13601988; doi:10.14814/phy2.71113)
Supplement: Supplementary file 1 [file PHY2-14-e71113-s008.pdf]

SUPPLEMENTARY APPENDIX 1. PROTOCOL FOR IMAGE ACQUISITION AND DATA ANALYSIS

This protocol describes standardized echocardiographic image acquisition and analysis procedures used to generate developmental reference values for neonatal, juvenile, and adult C57BL/6 mice.

Image Acquisition

The echocardiography machine is a Vevo F2 ultrasound system with color and tissue Doppler from VisualSonics (http://www.visualsonics.com/). The transducer used depended on the mouse age and weight being echoed, for P0 – P21, UHF71x (71-30 MHz), and for adult mice UHF57x (57-25 MHz).

- Select the preset to neonatal cardiac (71 MHz) or cardiac (57 MHz) based on the probe used.

Note on P0 imaging: At postnatal day 0 (P0), no anesthesia or hair removal is performed. Pups are gently restrained with surgical tape on the heated platform. Imaging at P0 is limited to parasternal LAX B-mode, SAX B-mode, and SAX M-mode. Doppler, tissue Doppler, strain analysis, LA area, and RV assessment are not performed at P0 due to the technical constraints of neonatal imaging at this stage.
Tip: When handling neonatal pups, rub gloved hands in the home cage bedding and nesting material before and after each handling session. This transfers familiar scent onto the gloves and minimizes foreign odor deposition on the pups, reducing the risk of maternal rejection upon return to the litter. This step is particularly important at P0 and P8 when pups are most vulnerable.

- Heat up echocardiography gel and turn on the small animal heat pad for 30 min before starting.
- Determine the body weight of the animal before initiating the echocardiography procedure.
- Anesthetize the animal with isoflurane (2-3%) in a chamber and then place mouse on the Vevo Imaging heated platform in the supine position and provide anesthesia via a nosecone. Titrate to maintain adequate sedation level (1-1.5 %).
  Tip: For smaller mice, adjust the nose cone appropriately so that the limbs are better aligned with the electrodes.
- Gently secure the mouse’s limbs to the platform using 3M surgical tape on the appropriate ECG lead positions on the stage and apply electrode gel to ensure appropriate heart rate

monitoring without artifact. Adjust anesthesia as necessary to maintain a target heart rate >400 beats per minute (bpm) during the echocardiography procedure.

Tip: To obtain readings in smaller mice (P8) that are not large enough to reach the electrodes, use a syringe filled with electrode gel and draw a line connecting the animal limbs to the electrodes.

- Monitor temperature to ensure normothermia.

  Tip: Neonatal mice are very sensitive to temperature changes and cannot use the temperature probe to monitor temperature. To avoid hypothermia, ensure the heated platform and gel warmer are turned on at least 30 minutes prior to the echocardiography procedure. Additionally, a heat lamp can be used as an extra heat source if needed.

- Apply depilatory cream to the chest area for approximately 2 minutes and then use warm wet gauze to wipe off excess depilatory cream and clean remaining hair.

  Tip: Apply another round of depilatory cream if hair patches remain but be cautious to not cause skin irritation.

- Apply pre-warmed echocardiography gel onto the chest.

  Tip: For better results, gel may be added in 50ml Eppendorf tubes and centrifuged prior to echocardiography in order to remove bubbles.

- Parasternal Long-Axis View (LAX) B-Mode: Keep the animal platform horizontal (avoid excessive tilting as this can cause changes in the hemodynamics), place the transducer over the sternum vertically with the notch pointing towards the base of the heart (in the direction of the animal's right shoulder) and obtain the two-dimensional (2D) parasternal long-axis view (LAX) in B-mode.

  Tip: Adjust the angle of the transducer to avoid rib/sternum shadows.

  Key landmarks:

  i. Left Ventricle (LV)
  ii. Aorta (in horizontal line with apex)
  iii. Left Atrium (LA)
  iv. Right Ventricular Outflow Tract (RVOT)

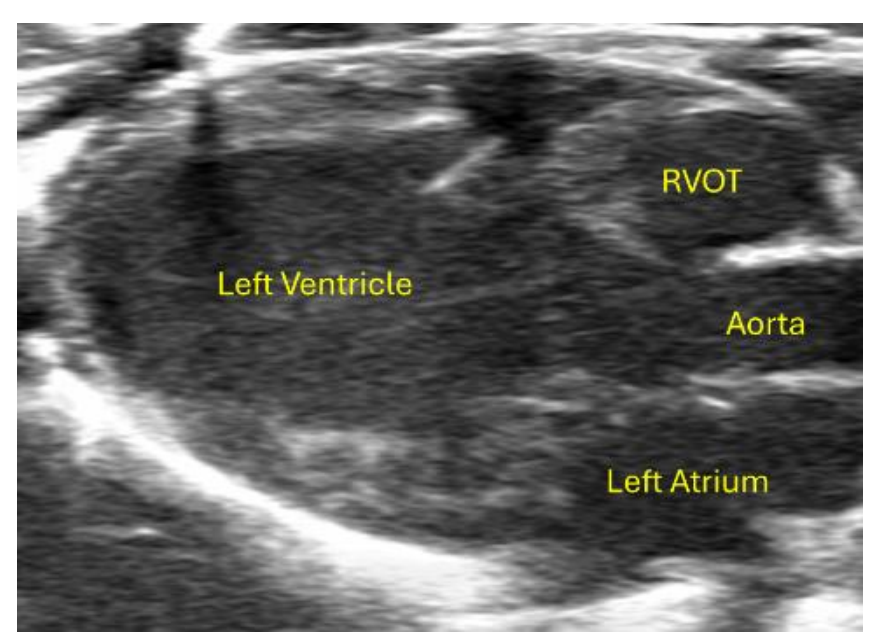

- Parasternal Short-Axis View (SAX) B-Mode: With the transducer positioned in the LAX view as mentioned above, rotate the transducer 90° clockwise to obtain the short-axis view (SAX) at the level of the papillary muscles.

Tip: Make slight Y-axis adjustments and/or tilt platform/probe accordingly so that the papillary muscles are aligned on the right side of the view at the appropriate mid-papillary level (2 and 4 o'clock) to decrease measurement variability.

Key landmarks:

i. LV
ii. RV
iii. Papillary muscles

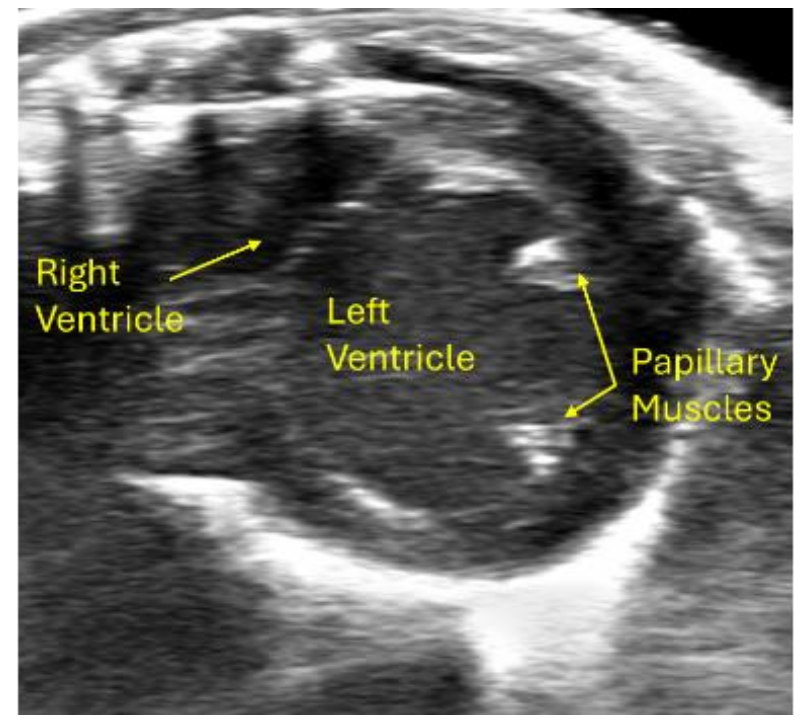

- Parasternal Short-Axis View (SAX) M-Mode: Once the papillary muscles are aligned appropriately, the M-mode sample volume is placed at the mid-level of the LV where the diameter is the greatest.

  Tip: Make sure the sample volume includes both anterior and posterior wall borders. Avoid papillary muscles in the sample as it may be confused with the posterior wall, resulting in falsely elevated EF values. Make fine adjustments in the Y- or X-axis plan to optimize the view and obtain clearer lines.

- Apical Four Chamber View B-Mode: To obtain the view, with the transducer positioned in the SAX view as mentioned above, tilt the transducer backwards at a 45-degree angle, while maintaining the same angle as the SAX view. In the 4-chamber view the ventricles will appear at the top of the image and the atria at the bottom.

  Tip: Pressing too hard on the chest may compress the atria, causing them to disappear and lead to inaccurate measurements. Additionally, caution should be applied while obtaining this measurement as extreme platform tilt or transducer pressure may cause hemodynamic instability and cardiac depression to the animal.

  Key landmarks:

  i. Left ventricle
  ii. Left atrium
  iii. Right ventricle
  iv. Right atrium
  v. Septum

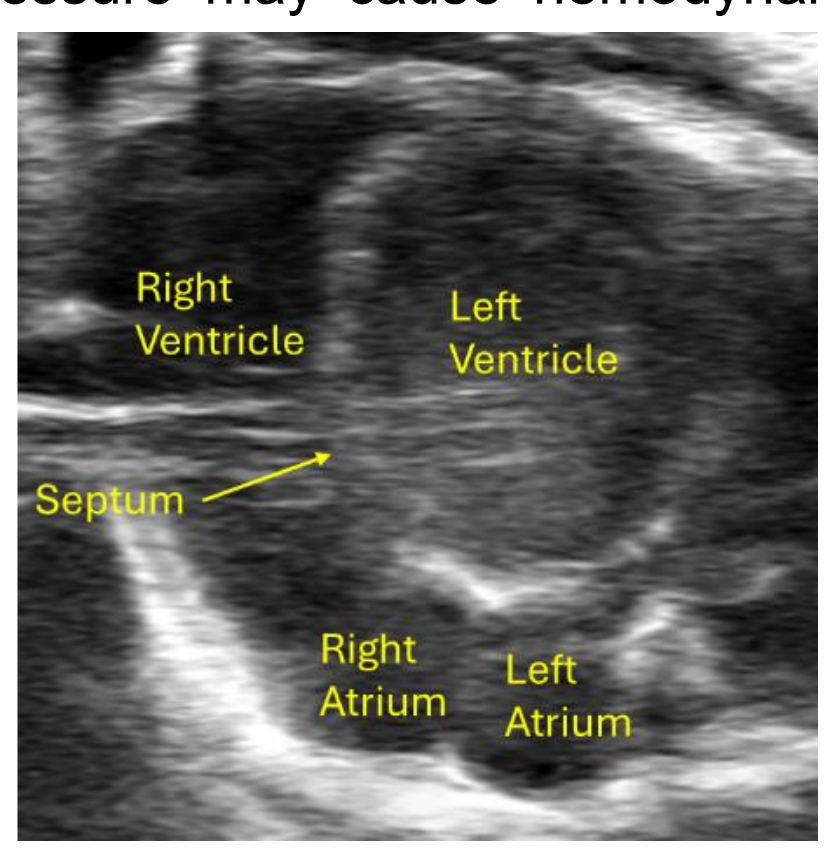

- Apical Four Chamber View Color Doppler: From the view above, switch to color doppler mode. Perform color doppler acquisition to assess continuous flow from the left atrium to the LV to confirm an appropriate view. To then assess diastolic function by mitral blood flow velocity,

obtain Pulse Wave (PW) Doppler. Center the PW doppler sample volume at the area with the highest blood flow velocity (brightest blue hues) within the LV chamber. Doppler angle should point towards the overall direction of the blood flow, usually aiming toward the apex, but should not go beyond 45 degrees.

Tip: If color Doppler signal is insufficient or rib cage and sternum shadowing obscures the PW Doppler signal, try minor adjustments in the Y- or X-axis plane, slight transducer rotation to find an acoustic window between ribs, gentle lateral pressure to shift the imaging plane away from bony structures, or adjusting the platform by a slight angle of 5–10 degrees. Note that the Doppler angle should not exceed 45 degrees relative to the direction of blood flow, as larger angles will underestimate true velocities.

- Apical Four Chamber View Tissue Doppler: For the tissue doppler, the sample should be placed at the annulus of the mitral valve on the septal wall side, at the intersection between the LV septum and the septal wall mitral valve. In mice, the mitral valve-free wall is usually obscured and unavailable for measurement.

  Tip: If there is an absence of a distinguishable a' wave, the animal may have a high heart rate. Adjust the anesthesia flow as necessary to reduce heart rate slightly without dropping below 400 bpm.
- Tricuspid annular plane systolic excursion (TAPSE): In the apical four chamber view place the M-mode sample volume across the tricuspid valve near its attachment to the LV free septal wall.

  Tip: Place the sample close to the tip of the leaflet to capture the clearest images. A septal wall TAPSE may also be measured.
- For all image acquisition, wait for the white loading bar above the ECG to finish before saving the image to obtain the maximum amount of frames set and length of cine loop.
- After successful recordings of desired measurements ensure removal of echocardiography gel, heating pad, and transducer. Release the mouse by removing the tape gently with an adhesion remover and place mouse back in the cage for monitoring until fully recovered.

Image Analysis

The echocardiography program used for analysis is Vevo LAB version 5.11.0 by VisualSonics (http://www.visualsonics.com/).

- LA Area: Measured from the B-Mode LAX as in our experience we found it more reliable compared to the four-chamber view. Maximum LA area (LAA systole) occurs at the end of ventricular systole, just before the mitral valve opens. This corresponds to the end of the T

wave on the ECG. Minimum LA area (LAA diastole) occurs at the end of ventricular diastole, immediately after the mitral valve closes. This corresponds to the QRS complex on the ECG. Tip: Use the slider under the image to move back and forth just a couple frames before making the measurement to better identify the outline of the atrium during systole and diastole.

- M-mode Analysis: Trace walls starting from a point in systole and include at least 2-3 cycles. Avoid cycles with distortions from respiration or animal movement as these will affect accuracy of readings. Make at least 3 separate measurements to be averaged.

  Tip: M-mode derived volumetric parameters (LVEDV, LVESV, EF, SV, CO) assume a spherical LV geometry (Teichholz formula). While reproducible and widely used, these estimates may overestimate true volumes compared to B-mode LAX methods, particularly in remodeled or diseased hearts. For healthy developmental reference values as presented here, this limitation is less critical but should be noted when applying these values to disease models.
- PW Doppler Analysis Mitral Valve Flow: Measure E, A, isovolumic contraction time (IVCT), aortic ejection time (AET), isovolumic relaxation time (IVRT), and mitral valve ejection time (MV ET) in this image. Make 3 sets of measurements, avoiding noise from breaths or animal movement.

  Tip: The magnification feature may be helpful for making more precise measurements.
- Tissue Doppler Analysis: Measure e', a', and s' wave, again making sure to avoid noise from breaths or animal movement. Make 3 sets of these measurements.

  Tip: The order of peaks in the PW Tissue is s wave, IVRT, e', a', and IVCT before looping back to the next s' wave. If one of either e' or a' appears to be missing, it is more likely that a' is missing since it represents the atrial kick.
- Tricuspid Annular Plane Systolic Excursion (TAPSE) Analysis: Measure the linear base-to-apex shortening of the RV during systole. Make at least 3 consecutive cardiac cycle measurements.

  Tip: Use the magnification tool to trace leaflet movement with more accuracy.
- Strain Measurement: Choose an ideal LAX B-mode image and open it in Vevo Strain 2.0. Make sure that the line drawn for the M-mode passes through the part of the LV where the diameter is greatest. Select as many cardiac cycles as possible without including noise from breaths or animal movement as this will skew data. When placing the systole and diastole markers, it is ideal that what can be visually seen as systole and diastole from the generated M-mode align with the ECG. If the ECG is present, there should already be period markers generated at end diastole. After drawing traces at the previously selected frames for end systole and diastole the program will automatically generate the results. Check to make sure

that the tracking is accurate. If it is not, repeat the trace from end systole or diastole depending on where the tracking issue seems to occur.

Tip: Period markers in the period selection step may be manually added for instances where the ECG was not available. During trace creation, drawing the endocardial border will automatically create the epicardial border. The size of the entire generated epicardial border can be increased or decreased using the arrows in the interface. Selecting either the endocardial or epicardial trace and SHIFT+S will apply a smoothing algorithm to the line, correcting any accidental sharp corners. CTRL+drag on a trace will allow movement of the entire trace at once which is often useful when going back to a trace to make fine adjustments.
