# Supplementary material for: Prospective echocardiographic characterization of postnatal cardiac development in C57BL/6 mice: Strain analysis between birth and adulthood
Source: Physiol Rep. 2026 Sep 24;14(18):e71113. doi: 10.14814/phy2.71113 (PMC13601988; doi:10.14814/phy2.71113)
Supplement: Supplementary file 2 [file PHY2-14-e71113-s009.docx]

**

**

**Supplemental Figure S1: Longitudinal Sex Differences in Strain.** (**a, b**) Line graphs illustrating developmental trajectories for (**a**) Endocardial Global Longitudinal Strain (EndoGLS) and (**b**) Global Radial Strain (GRS) in male (blue) and female (pink) mice from P8 to Wk8. Data are presented as mean ± SEM. P-values derived from independent samples t-tests comparing males vs. females at each time point. Significance: ** p < 0.01.

**Supplemental Figure S2: Bland-Altman Analysis Comparing M-mode and B-mode Derived Ejection Fraction Across Postnatal Development.** The difference between B-mode (Simpson's monoplane) and M-mode (Teichholz formula) derived ejection fraction (EF) is plotted against the mean of the two methods for each animal at each developmental timepoint. The solid black horizontal line represents the mean bias (-12.3%), indicating that M-mode systematically overestimates EF relative to B-mode across all timepoints. Red dashed lines indicate the 95% limits of agreement (ULoA: +2.9%; LLoA: -27.4%). Data points are color- and shape-coded by developmental timepoint: P0 (orange circles), P8 (green squares), P21 (blue triangles), and Wk8 (pink diamonds).

**Supplemental Figure S3: Echocardiography Reference Quicksheet.** Summary of key reference values derived from Table 1, formatted for use as a bench-side reference during image acquisition and analysis.

| Supplemental Table S1: Statistical Analysis of Sex-Specific Differences in Postnatal Cardiac Development | | | |
| --- | --- | --- | --- |
| Parameter | **P-value (Time)** | **P-value (Sex)** | **P-value (Time × Sex Interaction)** |
| BW (g) | < 0.001*** | < 0.001*** | < 0.001*** |
| Short Axis M-mode |  |  |  |
| HR (bpm) | < 0.001*** | 0.063 | 0.904 |
| dLVD (mm) | < 0.001*** | 0.405 | 0.580 |
| sLVD (mm) | < 0.001*** | 0.129 | 0.937 |
| dLVAW (mm) | < 0.001*** | 0.517 | 0.411 |
| sLVAW (mm) | < 0.001*** | 0.914 | 0.905 |
| dLVPW (mm) | < 0.001*** | 0.804 | 0.471 |
| sLVPW (mm) | < 0.001*** | 0.464 | 0.099 |
| LVEDV (µL) | < 0.001*** | 0.643 | 0.503 |
| LVESV (µL) | < 0.001*** | 0.233 | 0.882 |
| EF (%) | < 0.001*** | 0.061 | 0.997 |
| FS (%) | 0.007** | 0.059 | 0.999 |
| SV (µL) | < 0.001*** | 0.393 | 0.043* |
| CO (mL/min) | < 0.001*** | 0.801 | 0.093 |
| Long Axis B-mode | | | |
| dLAA (mm^2^) | < 0.001*** | 0.921 | 0.279 |
| sLAA (mm^2^) | < 0.001*** | 0.548 | 0.441 |
| FAC (%) | 0.011* | 0.383 | 0.177 |
| Mitral Valve Doppler |  |  |  |
| a’ (mm/s) | < 0.001*** | 0.529 | 0.059 |
| e’ (mm/s) | < 0.001*** | 0.354 | 0.687 |
| s’ (mm/s) | < 0.001*** | 0.009** | 0.002** |
| AET (ms) | 0.058 | 0.075 | 0.626 |
| IVCT (ms) | 0.007** | 0.659 | 0.124 |
| IVRT (ms) | 0.056 | 0.633 | 0.717 |
| ET (ms) | < 0.001*** | 0.035* | 0.891 |
| A (mm/s) | < 0.001*** | 0.441 | 0.806 |
| E (mm/s) | < 0.001*** | 0.523 | 0.937 |
| E/A | < 0.001*** | 0.440 | 0.641 |
| E/e’ | < 0.001*** | 0.981 | 0.756 |
| RV Function |  |  |  |
| TAPSE (mm) | < 0.001*** | 0.421 | 0.268 |
| Strain |  |  |  |
| MyoGCS (%) | 0.103 | 0.455 | 0.380 |
| MyoGLS (%) | < 0.001*** | 0.354 | 0.067 |
| EndoGCS (%) | 0.468 | 0.542 | 0.139 |
| EndoGLS (%) | 0.026* | 0.193 | 0.010* |
| GRS (%) | 0.024* | 0.473 | 0.002** |
| P-values determined by two-way mixed ANOVA assessing the main effects of Time (Age) and Sex, and their interaction (Time × Sex). Significant Time × Sex interactions (p<0.05) indicate developmental trajectories that diverge between males and females Significance is denoted as follows: * p < 0.05, ** p < 0.01, *** p < 0.001. Abbreviations: BW, body weight; HR, heart rate; LVD, left ventricular diameter; LVAW, left ventricular anterior wall thickness; LVPW, left ventricular posterior wall thickness; LVEDV, left ventricular end-diastolic volume; LVESV, left ventricular end-systolic volume; EF, left ventricular ejection fraction (Teichholz); FS, fractional shortening; SV, stroke volume; CO, cardiac output; LAA, left atrial area; FAC, left atrial fractional area change; a’, peak late-diastolic annular velocity; e’, peak early-diastolic annular velocity; s’, s-wave; AET, aortic ejection time; IVCT, isovolumic contraction time; IVRT, isovolumic relaxation time; ET, mitral valve ejection time; A, late diastolic transmitral flow velocity; E, early diastolic transmitral flow velocity; TAPSE, tricuspid annulus plane systolic excursion; MyoGCS, myocardial global circumferential strain; MyoGLS, myocardial global longitudinal strain; EndoGCS, endocardial global circumferential strain; EndoGLS, endocardial global longitudinal strain; GRS, global radial strain. The prefixes 'd' and 's' denote diastole and systole, respectively. | | | |

| Supplemental Table S2: Sex-Specific Comparisons at Each Developmental Time Point | | | | |
| --- | --- | --- | --- | --- |
| Parameter | **P0 (n=30)** | **P8 (n=28)** | **P21 (n=26)** | **Wk8 (n=26)** |
| BW (g) | 0.172 | 0.472 | 0.551 | < 0.001*** |
| Short Axis M-mode |  |  |  |  |
| HR (bpm) | 0.219 | 0.280 | 0.209 | 0.803 |
| dLVD (mm) | 0.378 | 0.458 | 0.364 | 0.589 |
| sLVD (mm) | 0.265 | 0.259 | 0.213 | 0.667 |
| dLVAW (mm) | 0.243 | 0.358 | 0.225 | 0.612 |
| sLVAW (mm) | 0.208 | 0.780 | 0.823 | 0.844 |
| dLVPW (mm) | 0.944 | 0.400 | 0.882 | 0.167 |
| sLVPW (mm) | 0.747 | 0.542 | 0.740 | 0.015* |
| LVEDV (µL) | 0.382 | 0.505 | 0.331 | 0.581 |
| LVESV (µL) | 0.253 | 0.303 | 0.177 | 0.682 |
| EF (%) | 0.206 | 0.188 | 0.229 | 0.339 |
| FS (%) | 0.216 | 0.178 | 0.240 | 0.328 |
| SV (µL) | 0.549 | 0.850 | 0.852 | 0.054 |
| CO (mL/min) | 0.280 | 0.529 | 0.339 | 0.212 |
| Long Axis B-mode | | | | |
| dLAA (mm^2^) |  | 0.950 | 0.263 | 0.372 |
| sLAA (mm^2^) |  | 0.661 | 0.863 | 0.323 |
| FAC (%) |  | 0.501 | 0.107 | 0.920 |
| Mitral Valve Doppler |  |  |  |  |
| a’ (mm/s) |  | 0.397 | 0.773 | 0.072 |
| e’ (mm/s) |  | 0.878 | 0.654 | 0.349 |
| s’ (mm/s) |  | 0.978 | 0.613 | 0.002** |
| AET (ms) |  | 0.468 | 0.342 | 0.042* |
| IVCT (ms) |  | 0.039* | 0.290 | 0.313 |
| IVRT (ms) |  | 0.784 | 0.758 | 0.508 |
| ET (ms) |  | 0.099 | 0.386 | 0.141 |
| A (mm/s) |  | 0.622 | 0.818 | 0.488 |
| E (mm/s) |  | 0.741 | 0.652 | 0.504 |
| E/A |  | 0.940 | 0.689 | 0.418 |
| E/e’ |  | 0.666 | 0.867 | 0.553 |
| RV Function |  |  |  |  |
| TAPSE (mm) |  | 0.500 | 0.652 | 0.233 |
| Strain |  |  |  |  |
| MyoGCS (%) |  | 0.603 | 0.548 | 0.233 |
| MyoGLS (%) |  | 0.498 | 0.944 | 0.019* |
| EndoGCS (%) |  | 0.846 | 0.376 | 0.133 |
| EndoGLS (%) |  | 0.793 | 0.745 | 0.002** |
| GRS (%) |  | 0.539 | 0.291 | 0.008** |
| P-values derived from independent samples t-tests comparing males vs. females at each time point. Significance: * p < 0.05, ** p < 0.01, *** p < 0.001. Abbreviations: BW, body weight; HR, heart rate; LVD, left ventricular diameter; LVAW, left ventricular anterior wall thickness; LVPW, left ventricular posterior wall thickness; LVEDV, left ventricular end-diastolic volume; LVESV, left ventricular end-systolic volume; EF, left ventricular ejection fraction (Teichholz); FS, fractional shortening; SV, stroke volume; CO, cardiac output; LAA, left atrial area; FAC, left atrial fractional area change; a’, peak late-diastolic annular velocity; e’, peak early-diastolic annular velocity; s’, s-wave; AET, aortic ejection time; IVCT, isovolumic contraction time; IVRT, isovolumic relaxation time; ET, mitral valve ejection time; A, late diastolic transmitral flow velocity; E, early diastolic transmitral flow velocity; TAPSE, tricuspid annulus plane systolic excursion; MyoGCS, myocardial global circumferential strain; MyoGLS, myocardial global longitudinal strain; EndoGCS, endocardial global circumferential strain; EndoGLS, endocardial global longitudinal strain; GRS, global radial strain. The prefixes 'd' and 's' denote diastole and systole, respectively. | | | | |

| Supplemental Table S3: Echocardiographic Measurement Feasibility | | | | |
| --- | --- | --- | --- | --- |
| Parameter | **P0 (n=30)** | **P8 (n=28)** | **P21 (n=26)** | **Wk8 (n=26)** |
| BW | 30/30 (100%) | 28/28 (100%) | 26/26 (100%) | 26/26 (100%) |
| Short Axis M-mode | | | | |
| HR | 30/30 (100%) | 28/28 (100%) | 26/26 (100%) | 26/26 (100%) |
| dLVD | 30/30 (100%) | 28/28 (100%) | 26/26 (100%) | 26/26 (100%) |
| sLVD | 30/30 (100%) | 28/28 (100%) | 26/26 (100%) | 26/26 (100%) |
| dLVAW | 30/30 (100%) | 28/28 (100%) | 26/26 (100%) | 26/26 (100%) |
| sLVAW | 30/30 (100%) | 28/28 (100%) | 26/26 (100%) | 26/26 (100%) |
| dLVPW | 30/30 (100%) | 28/28 (100%) | 26/26 (100%) | 26/26 (100%) |
| sLVPW | 30/30 (100%) | 28/28 (100%) | 26/26 (100%) | 26/26 (100%) |
| LVEDV | 30/30 (100%) | 28/28 (100%) | 26/26 (100%) | 26/26 (100%) |
| LVESV | 30/30 (100%) | 28/28 (100%) | 26/26 (100%) | 26/26 (100%) |
| EF | 30/30 (100%) | 28/28 (100%) | 26/26 (100%) | 26/26 (100%) |
| FS | 30/30 (100%) | 28/28 (100%) | 26/26 (100%) | 26/26 (100%) |
| SV | 30/30 (100%) | 28/28 (100%) | 26/26 (100%) | 26/26 (100%) |
| CO | 30/30 (100%) | 28/28 (100%) | 26/26 (100%) | 26/26 (100%) |
| Long Axis B-mode | | | | |
| dLAA |  | 22/28 (79%) | 20/26 (77%) | 23/26 (88%) |
| sLAA |  | 22/28 (79%) | 20/26 (77%) | 23/26 (88%) |
| FAC |  | 22/28 (79%) | 20/26 (77%) | 23/26 (88%) |
| Mitral Valve Doppler | | | | |
| a’ |  | 28/28 (100%) | 25/26 (96%) | 24/26 (92%) |
| e’ |  | 28/28 (100%) | 25/26 (96%) | 26/26 (100%) |
| s’ |  | 28/28 (100%) | 25/26 (96%) | 26/26 (100%) |
| AET |  | 28/28 (100%) | 25/26 (96%) | 26/26 (100%) |
| IVCT |  | 28/28 (100%) | 25/26 (96%) | 26/26 (100%) |
| IVRT |  | 28/28 (100%) | 25/26 (96%) | 26/26 (100%) |
| ET |  | 28/28 (100%) | 25/26 (96%) | 26/26 (100%) |
| A |  | 27/28 (96%) | 25/26 (96%) | 23/26 (88%) |
| E |  | 28/28 (100%) | 25/26 (96%) | 26/26 (100%) |
| E/A |  | 27/28 (96%) | 25/26 (96%) | 23/26 (88%) |
| E/e’ |  | 28/28 (100%) | 25/26 (96%) | 26/26 (100%) |
| RV Function | | | | |
| TAPSE |  | 28/28 (100%) | 25/26 (96%) | 26/26 (100%) |
| Strain | | | | |
| MyoGCS |  | 28/28 (100%) | 26/26 (100%) | 26/26 (100%) |
| MyoGLS |  | 28/28 (100%) | 26/26 (100%) | 26/26 (100%) |
| EndoGCS |  | 28/28 (100%) | 26/26 (100%) | 26/26 (100%) |
| EndoGLS |  | 28/28 (100%) | 26/26 (100%) | 26/26 (100%) |
| GRS |  | 28/28 (100%) | 26/26 (100%) | 26/26 (100%) |
| Echocardiographic measurement feasibility percentages for each time point. Abbreviations: BW, body weight; HR, heart rate; LVD, left ventricular diameter; LVAW, left ventricular anterior wall thickness; LVPW, left ventricular posterior wall thickness; LVEDV, left ventricular end-diastolic volume; LVESV, left ventricular end-systolic volume; EF, left ventricular ejection fraction (Teichholz); FS, fractional shortening; SV, stroke volume; CO, cardiac output; LAA, left atrial area; FAC, left atrial fractional area change; a’, peak late-diastolic annular velocity; e’, peak early-diastolic annular velocity; s’, s-wave; AET, aortic ejection time; IVCT, isovolumic contraction time; IVRT, isovolumic relaxation time; ET, mitral valve ejection time; A, late diastolic transmitral flow velocity; E, early diastolic transmitral flow velocity; TAPSE, tricuspid annulus plane systolic excursion; MyoGCS, myocardial global circumferential strain; MyoGLS, myocardial global longitudinal strain; EndoGCS, endocardial global circumferential strain; EndoGLS, endocardial global longitudinal strain; GRS, global radial strain. The prefixes 'd' and 's' denote diastole and systole, respectively. | | | | |
